# Profiling Localized Immunomodulation and Drug Biodistribution within a Subcutaneous Vascularized Niche for Cell Transplantation

**DOI:** 10.1002/advs.202520914

**Published:** 2026-03-06

**Authors:** Jocelyn Nikita Campa‐Carranza, Simone Capuani, Melissa A. Willman, Alexander Rabassa, Ashley L. Joubert, Tommaso Bo, Letizia Franco, Marzia Conte, Ana L. Anaya‐García, Gabrielle E. Rome, Rim Ouni, Henry J. Seaborne, Camden A. Caffey, Dora M. Berman, Junjun Zheng, Jesus Paez‐Mayorga, Corrine Ying Xuan Chua, Shu Hsia Chen, Norma S. Kenyon, Alessandro Grattoni

**Affiliations:** ^1^ Center for BioNanoengineering Houston Methodist Research Institute Houston Texas USA; ^2^ School of Medicine and Health Sciences Tecnologico de Monterrey Monterrey Nuevo León Mexico; ^3^ Diabetes Research Institute University of Miami Miami Florida USA; ^4^ Department of Applied Science and Technology Politecnico di Torino Torino Italy; ^5^ Center for Immunotherapy Research Houston Methodist Research Institute Houston Texas USA; ^6^ Immunomonitoring Core Houston Methodist Research Institute Houston Texas USA; ^7^ Department of Surgery Miller School of Medicine University of Miami Miami Florida USA; ^8^ Department of Microbiology and Immunology Miller School of Medicine University of Miami Miami Florida USA; ^9^ Department of Biomedical Engineering University of Miami Miami Florida USA; ^10^ Department of Biochemistry and Molecular Biology University of Miami Miami Florida USA; ^11^ Department of Surgery Houston Methodist Hospital Houston Texas USA; ^12^ Department of Radiation Oncology Houston Methodist Hospital Houston Texas USA

**Keywords:** cell encapsulation, cell therapy, drug biodistribution, immunomodulation, islet transplantation, localized immunosuppression, pharmacokinetics

## Abstract

Systemic immunosuppression remains essential for preventing allogeneic transplant rejection, but its chronic use causes substantial toxicity. Conceptually, local, site‐specific immunomodulation offers a promising alternative, yet comparative mechanistic insight into how immunosuppressants behave when delivered directly to the graft is lacking. Here, we leveraged the Neovascularized Implantable Cell Homing and Encapsulation (NICHE) device, a subcutaneous, vascularized cell‐encapsulation platform, as a spatially defined and reproducible model to study local immunomodulation in allogeneic islet transplantation. We systematically profiled the safety, local and systemic immunomodulatory effects, pharmacokinetics, and longitudinal biodistribution of five clinically relevant agents delivered locally at the graft site: CTLA4‐Ig, anti‐lymphocyte serum, anti‐CD40L, anti‐CD2, and anti‐IL6. Sustained in situ exposure did not impair islet viability or function, and immunosuppressants were confined within the graft, with up to 100‐fold lower systemic concentrations. Individual agents produced distinct immune signatures, spanning lymphocyte depletion and shifts in T‐cell activation that can guide rational, mechanism‐informed combinations for allogeneic cell transplantation. These findings provide a comparative framework for evaluating localized immunosuppression with the potential to transform immunoprotection in cell therapy.

## Graphical abstract

We studied how five common immunosuppressants behave when delivered directly to a transplant site instead of systemically. Using a vascularized implant for islet transplantation, we show that local delivery protects grafts, limits drug spread to the rest of the body, and produces distinct immune signatures. Defining these specific signatures enables the development of more effective combinations with reduced adverse effects.

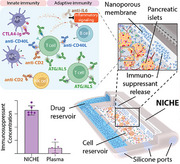

## 1 Introduction

The success of allogeneic islet transplantation as a functional cure for type 1 diabetes (T1D) depends on preventing immune rejection of the graft while maintaining a local microenvironment that supports cell survival and function. Systemic immunosuppression remains the clinical standard for transplant protection; however, its chronic use imposes a significant burden on patients, who must adhere to lifelong daily medication and face risks of adverse events, including graft toxicity [1, 2], opportunistic infections [3], and associated malignancies [4, 5], This dependency impacts quality of life, limits broader applicability of the approach and poses patient adherence challenges [6], whether islets are delivered intrahepatically or via encapsulation devices that still rely on systemic immunosuppression [7, 8]. Furthermore, systemic administration of immunosuppressants (IS) often fails to achieve effective concentrations at the graft site while exposing off‐target organs to high drug levels, contributing to cumulative toxicity [9, 10]. These limitations underscore the need for site‐specific immunomodulatory strategies that minimize systemic exposure while preserving islet viability.

Despite widespread clinical use of IS in organ transplantation, there is no consensus on an optimal immunosuppressive regimen for islet transplantation. Most protocols combine lymphocyte‐depleting induction therapies with chronic maintenance regimens to prevent chronic rejection [11, 12]. Yet systemic delivery remains inherently non‐specific. Local immunosuppressive strategies offer a promising alternative [13, 14, 15], particularly in accessible subcutaneous transplantation platforms, where local long‐acting delivery is feasible and can provide long‐term graft protection while minimizing systemic drug exposure. Given the complexity of the alloimmune cascade, a rational, multitargeted approach is likely required to achieve durable protection [16]. However, it is unclear how individual IS perform when delivered locally, and whether their systemic mechanism of action translates to a confined transplant site. Addressing this knowledge gap is fundamental for the rational design of localized immunomodulatory regimens.

**FIGURE 1:**
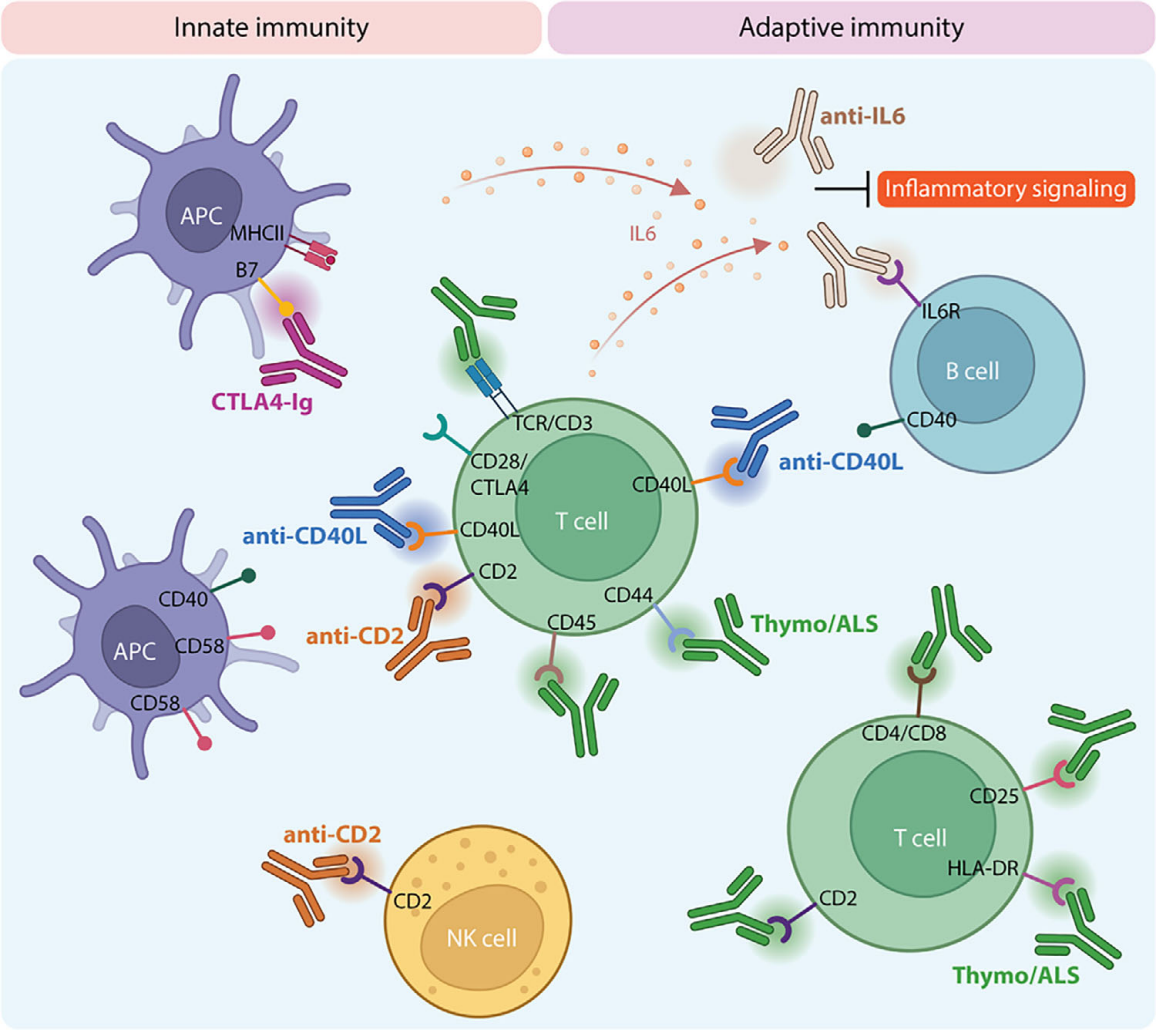
Schematic illustration of the mechanistic targets of clinically relevant IS agents for use in a local setting. Overview of the sites of action for CTLA4‐Ig (purple), Thymo/ALS (green), anti‐CD40L (blue), anti‐CD2 (orange), and anti‐IL6 (brown) with downstream effects on to CD28/CTLA4 mediated co‐stimulation (CTLA4‐Ig), T cell depletion (Thymo/ALS), CD40‐CD40L co‐stimulation (anti‐CD40L), CD2/CD58 mediated adhesion (anti‐CD2), and IL‐6 inflammatory signaling and B cell activation (anti‐IL6).

Because local delivery produces sustained drug exposure within the graft, we prioritized agents with evidence for immunomodulatory efficacy without direct islet toxicity observed with calcineurin inhibitors and mTOR inhibitors, which have been reported to impair insulin secretion, β‐cell survival, and islet engraftment. To this end, we studied five clinically relevant immunosuppressive agents targeting complementary nodes of transplant rejection (Figure 1): CTLA4‐Ig (Abatacept, Belatacept), anti‐lymphocyte serum (ALS, analog of Thymoglobulin), anti‐CD40L, anti‐CD2, and anti‐IL6. These agents are either FDA‐approved or currently under clinical investigation for transplant applications. Their selection was based on complementary mechanisms of action, including T cell co‐stimulation blockade (CTLA4‐Ig, anti‐CD40L), lymphocyte depletion (ALS, anti‐CD2), cytokine signaling interference (anti‐IL6), and modulation of antigen‐presenting cell (APC) activity (anti‐CD40L anti‐CD2).

CTLA4‐Ig binds to B7‐1 (CD80) and B7‐2 (CD86) receptors on APCs, thereby blocking CD28‐mediated T cell co‐stimulation [17]. Belatacept is approved for kidney transplantation [18] and has shown promise in promoting islet allograft survival [19, 20]. Thymoglobulin is widely used for induction therapy in renal [21] and islet transplantation [12, 22, 23]. However, its systemic administration is typically limited to induction due to risks of cytokine release syndrome and higher susceptibility to infections [24, 25]. Anti‐CD40L disrupts the CD40‐CD40L and CD11b‐CD40L pathways, suppressing pro‐inflammatory cytokine production, and inhibiting effector T and B cell activation, while promoting T‐reg expansion [26]. Additionally, CD11b blockade reduces myeloid cell recruitment [27]. Though early clinical trials of anti‐CD40L were halted due to thromboembolic events [28], engineered variants that successfully avoid platelet activation have shown preclinical efficacy [29], renewing interest in this agent as a promising IS for organ transplantation. Anti‐CD2 (Siplizumab) depletes effector T cells, blocks co‐stimulation, and increases donor‐specific regulatory cells (Tregs) [30, 31]. CD2 is a surface adhesion and co‐stimulatory receptor expressed on T cells and NK cells, where it facilitates stable interactions with APCs via binding to CD58 (LFA‐3), enhancing T cell activation and cytotoxic function [32]. Thus, blockade of CD2‐CD58 interactions has been leveraged in tolerance‐inducing regimens, enabling withdrawal from maintenance immunosuppression in a subset of kidney transplant recipients [33]. Finally, IL‐6 is a pleiotropic cytokine known to promote inflammation, effector T cell differentiation, and B cell activation, making it a compelling but complex target for modulating alloimmune responses [34]. IL6 pathway blockade is under investigation for treatment of chronic active antibody‐mediated rejection (caAMR) [35], as it reduces donor‐specific antibody (DSA) production and inflammatory signaling.

To assess the localized effects of these IS agents in allogenic islet transplantation, we leveraged the Neovascularized Implantable Cell Homing and Encapsulation (NICHE) device, a subcutaneously implantable dual‐reservoir cell encapsulation platform [36, 37, 38, 39, 40], as a reproducible testbed. This system enables co‐delivery of IS agents and pancreatic islets within a defined subcutaneous space, allowing parallel evaluation of drug biodistribution, islet function, and the local and systemic immune response. We first established safe and immunologically active drug concentrations via in vitro immune proliferation assays, perifusion glucose‐stimulated insulin secretion (GSIS), and islet viability. We then performed in vivo studies to characterize drug biodistribution and immune cell dynamics across multiple compartments. Collectively, this comparative study reveals agent‐specific local immune fingerprints and demonstrates that sustained local delivery can maintain islet viability and function while limiting systemic exposure, evidence that is needed to design rational, multi‐targeted local regimens for cell therapy.

## 2 Results

### 2.1 In Vitro Immunosuppressive Activity and Islet Compatibility

Because preclinical and clinical data on the direct delivery of immunosuppressive agents at the transplantation site are scarce, we first sought to define concentrations that are both effective and non‐toxic to grafted cells and surrounding host tissues. The impact of IS on T cell suppression was assessed using rat allogeneic cell trace violet (CTV)‐labeled mixed lymphocyte reactions (MLRs). Cell proliferation was evaluated based on CTV dilution (Figure S1) across major B cells, NK cells, T cells, and their CD4^+^ and CD8^+^ subsets. Results are shown both as proliferation changes and total responder cell abundance (blue‐red gradient), the latter of which is relevant for interpreting ALS‐induced effects. In parallel, islet function and viability were evaluated after a five‐day incubation with each IS drug through perifusion GSIS assays and live/dead staining. These in vitro studies informed both lower effective and upper safety thresholds and served for critical validation of species‐specific bioactivity prior to in vivo use, particularly for anti‐CD40L (mouse‐specific), anti‐CD2, and anti‐IL6 (NHP‐specific).

**FIGURE 2:**
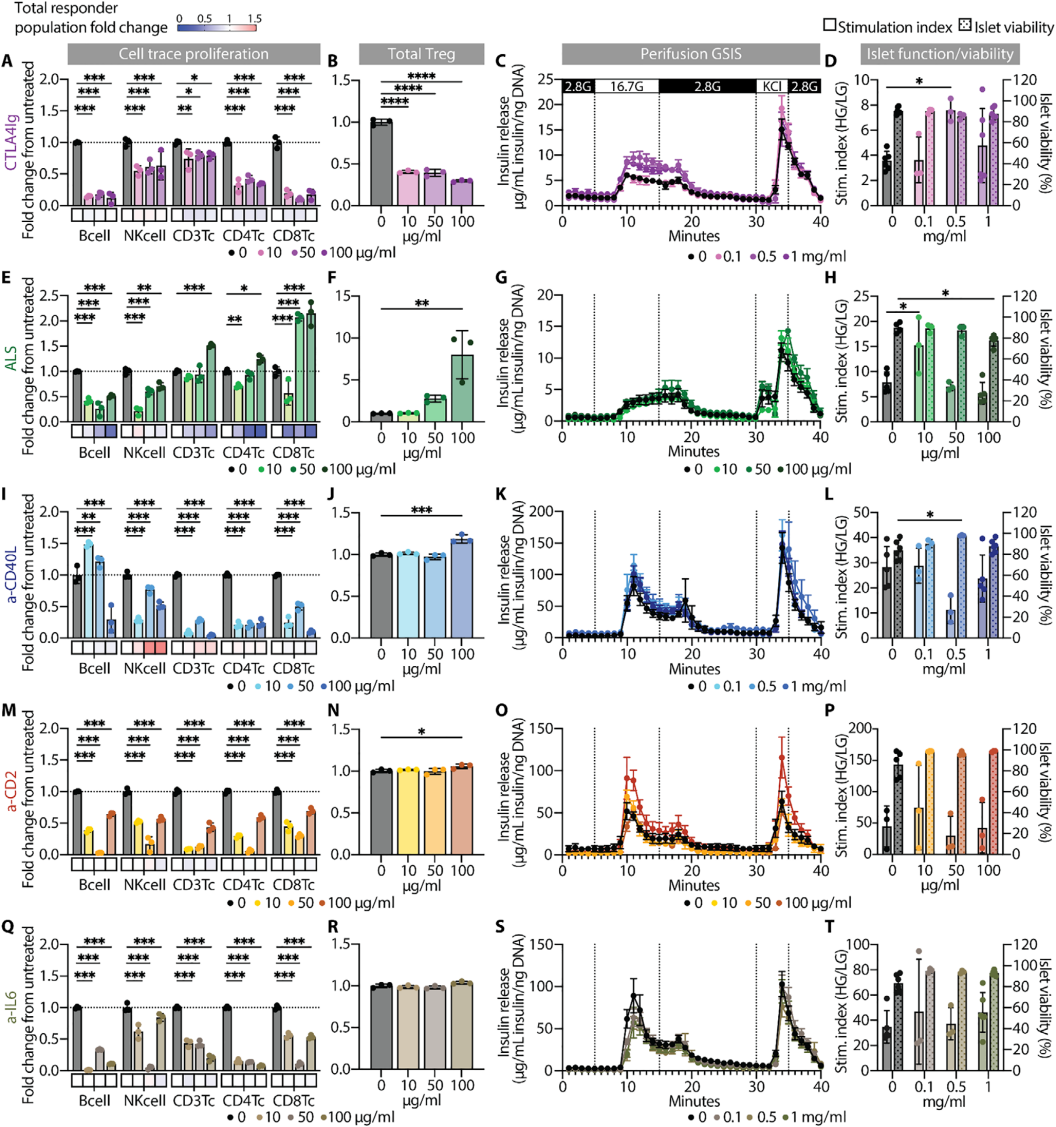
In vitro immunosuppressive activity and islet compatibility rodent assays. (A, E, I, M, Q) Proliferation profile of immune cell populations based on CTV dilution with complementary changes in total CTV‐labeled responder cells and (B, F, J, N, R) total Treg frequencies represented as fold change relative to untreated control (0 µg ml^−1^); (C, G, K, O, S) insulin release curves from perifusion GSIS and (D, H, L, P, T) associated stimulation index with % viability of pancreatic islets incubated in the presence or absence of IS. In vitro immunosuppressive effect was tested for CTLA4‐Ig at 10, 50, 100 µg mL^−1^ (A‐B), ALS at 10, 50, and 100 µg mL^−1^ (E‐F), anti‐CD40L at 10, 50, and 100 µg mL^−1^ (I‐L), anti‐CD2 at 10, 50, and 100 µg mL^−1^ (M‐N), and anti‐IL6 at 10, 50, and 100 µg mL^−1^ (Q‐R). Islet compatibility was tested for CTLA4‐Ig at 0.1, 0.5, 1 mg mL^−1^ (C‐D), ALS at 10, 50, and 100 µg mL^−1^ (G‐H), anti‐CD40L at 0.1, 0.5, 1 mg mL^−1^ (K‐L), anti‐CD2 at 10, 50, and 100 µg mL^−1^ (O‐P), and anti‐IL6 at 0.1, 0.5, 1 mg mL^−1^ (S‐T). All data presented for MLR (n = 3/treatment), GSIS, and live/dead (n = 3‐5/treatment) as mean ± SD. Drug concentration‐dependent changes were independently assessed for each cell population using one‐way ANOVA with Tukey's multiple comparisons test. (^*^ *p* < 0.05; ^**^ *p* < 0.01; ^***^ *p* < 0.001).

We previously demonstrated the long‐term efficacy of locally delivered CTLA4‐Ig and ALS in supporting allogeneic islet survival within the NICHE microenvironment [37]. In this study, CTLA4‐Ig suppressed proliferation across all lymphoid subsets, including B, NK, CD4^+^, and CD8^+^ T cells (Figure 2A), while regulatory T cells were decreased across all tested concentrations (10‐100 µg mL^−1^) (Figure 2B). Treated islets retained normal GSIS profiles, with a higher stimulation index observed at 0.5 mg mL^−1^ and no impact on viability (Figure 2C,D). These data support a broad safe dose range up to 1 mg mL^−1^ for local use, albeit at the expense of Treg depletion. ALS‐mediated depletion of the total responder lymphocyte population resulted in an apparent increase in T cell proliferation (Figure 2E). However, the absolute number of proliferating T cells did not increase, indicating no true proliferative response. Notably, at 100 µg mL^−1^ ALS produced an apparent enrichment of Tregs (Figure 2F). Yet, the absolute Treg numbers remained unchanged, consistent with post‐depletion regulatory skewing [41], rather than true expansion. Islet assays showed preserved GSIS function at 10–50 µg mL^−1^, with reduced viability observed at 100 µg mL^−1^ (Figure 2G,H), establishing a toxicity threshold of 50 µg mL^−1^.

Anti‐CD40L (clone MR‐1) suppressed T cell proliferation, with reduced B cell proliferation only at 100 µg mL^−1^ (Figure 2I), while Treg frequencies increased at higher concentrations (Figure 2J), confirming cross‐reactivity in the rat model. Interestingly, a relative increase in total NK cell responder abundance was observed despite reduced proliferation, which may reflect a compensatory response to the suppression of other lymphoid populations. Islet function and viability were unaffected with MR‐1 concentrations of up to 1 mg mL^−1^ (Figure 2K,L). Anti‐CD2 suppressed the proliferation of NK and T cells (Figure 2M), with minimal effect on Treg populations (Figure 2N). Islet function and viability were unaffected (Figure 2O,P), validating its suitability for local delivery in rodents. Similarly, anti‐IL6 exhibited comparable suppression to anti‐CD2 (Figure 2Q), preserved Tregs (Figure 2S), and showed no GSIS or viability impairment up to 1 mg mL^−1^ (Figure 2T,U).

Together, these in vitro assays confirmed species‐specific cross‐reactivity, identified agent‐specific immunomodulatory profiles, and established safe dose ranges to guide subsequent in vivo delivery via the NICHE platform.

### 2.2 Drug Biodistribution from the NICHE Device

**FIGURE 3:**
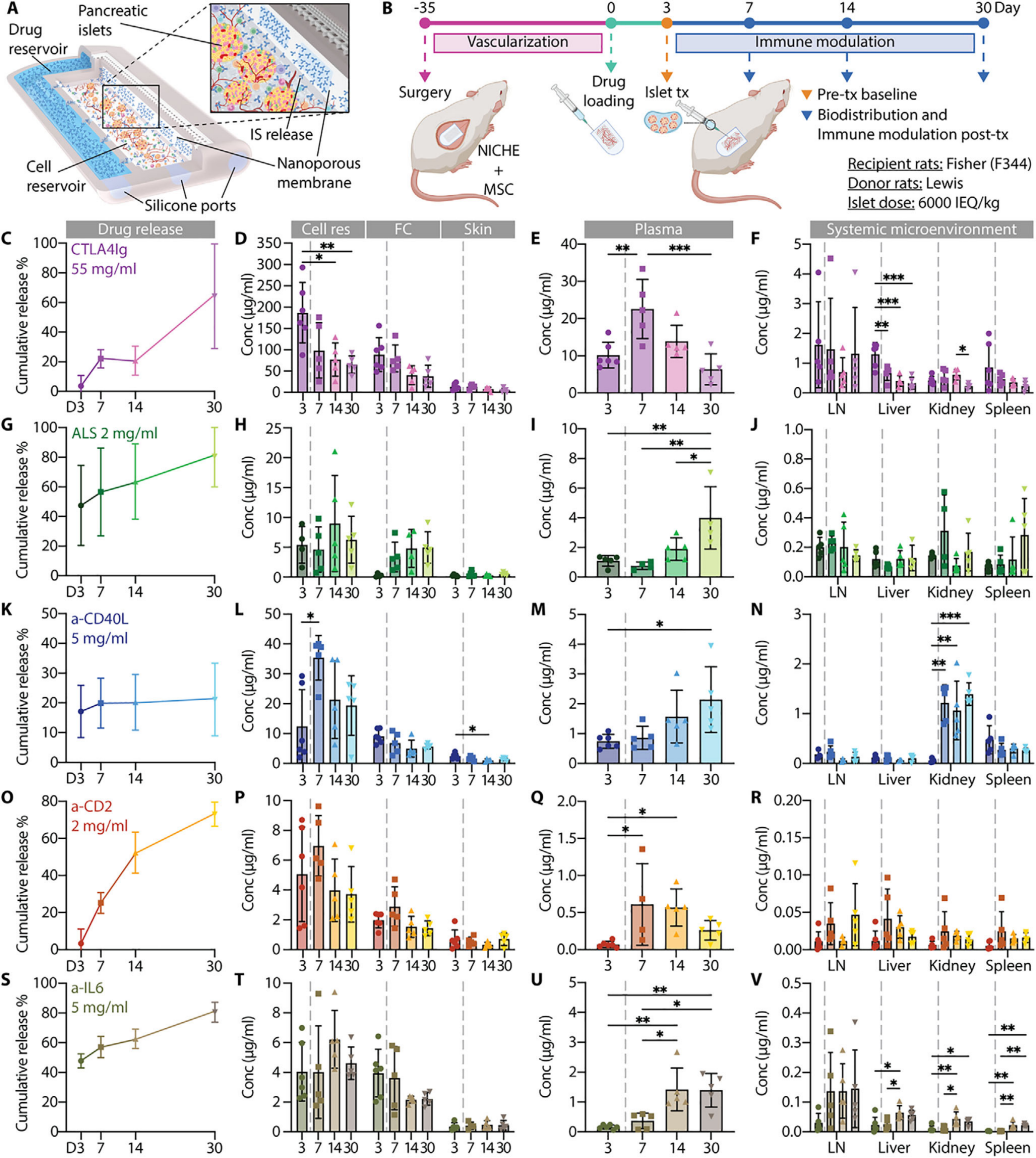
In vivo drug biodistribution. (A) 3D schematic of a NICHE device and its components. (B) Study design for IS biodistribution and immunomodulation assessment in the context of subcutaneous allogeneic pancreatic islet transplantation. (C, G, K, O, P) Calculated cumulative release, (D, H, L, P, T) distribution within the cell reservoir (cell res), surrounding fibrotic capsule (FC) and skin, (E, I, M, Q, U) plasma and (F, J, N, R, V) systemic dissemination of CTLA4Ig (C‐F), ALS (G‐J), anti‐CD40L (K‐N), anti‐CD2 (O‐R), and anti‐IL6 (S‐V) delivered locally via NICHE. N = 4‐6 for cumulative release calculation. All results are presented as mean ± SD. Time‐dependent changes within each tissue compartment were independently assessed using one‐way ANOVA with Tukey's multiple comparisons test. (^*^ *p* < 0.05; ^**^ *p* < 0.01; ^***^ *p* < 0.001).

The NICHE platform is an implantable, 3D‐printed cell encapsulation device comprising a vascularized cell reservoir for transplanted cells, surrounded by a drug reservoir. The drug reservoir is loaded with an immunosuppressive (IS) solution and delivers it by concentration‐driven diffusion through a nanoporous membrane into the central cell chamber, providing sustained local immune protection while limiting systemic exposure (Figure 3A). In previous studies, we showed that NICHE enables sustained, localized IS delivery directly at the transplant site [36, 37, 38]. Here, NICHE was used to investigate the spatial distribution, release kinetics, and immunomodulatory effects of clinically relevant IS agents within the transplant microenvironment, leveraging three core attributes: (1) precise in situ delivery with minimal systemic dissemination; (2) reproducible, sustained local release enabling consistent and comparative IS evaluation; and (3) a controlled environment for unequivocal quantification of local drug concentrations, allowing direct correlation with immunomodulatory efficacy.

NICHE devices loaded with a mesenchymal stem cells (MSCs)‐laden hydrogel, previously shown to enhance intradevice vascularization [40, 42], were implanted subcutaneously in rats. After 5 weeks the drug reservoir was transcutaneously loaded with one of five clinically relevant immunosuppressive (IS) agents: CTLA4‐Ig, ALS, anti‐CD40L, anti‐CD2, or anti‐IL6. All drugs were confirmed to be stable at 37°C for at least 8 weeks in conditions mimicking the ones of an implanted NICHE drug reservoir (Figure S2). Three days post IS loading, allogeneic pancreatic islets were transplanted into the cell reservoir. Based on our prior work [39, 40, 43], this schedule allowed sufficient vascular ingrowth within the cell reservois, creating an oxygen‐rich microenvironment that supports islet engraftment, viability, and function, while ensuring homogeneous IS distribution within the reservoir [38]. This configuration was used to assess both tissue‐specific biodistribution and systemic immunomodulatory effects of the IS agents over 30 days (Figure 3B). Biodistribution was quantified in NICHE compartments (cell reservoir, fibrotic capsule, and overlying skin), plasma, and major organs (draining lymph nodes, liver, kidney, and spleen). Assessment of drug concentration at day 3 post‐drug loading served to characterize pre‐transplant IS levels, whereas subsequent timepoints (days 7, 14, and 30) were used for the longitudinal assessment of biodistribution post‐transplant.

CTLA4‐Ig exhibited approximately steady release kinetics, (Figure 3C) initially accumulating locally (Figure 3D) and subsequently distributing systemically, reaching peak concentration in plasma of 22.56±7.96 µg mL^−1^ around day 7 (Figure 3E). Overall, plasma levels of CTLA4Ig were 10‐times lower than those in the cell reservoir (13.25 µg mL^−1^ vs. 107.01 µg mL^−1^) with limited but detectable distribution to systemic organs, most notably in the lymph nodes at earlier timepoints (max 1.62 µg mL^−1^) (Figure 3F). ALS cumulative release showed an initial burst followed by slower steady kinetics (Figure 3G), resulting in relatively stable concentrations in the local microenvironment across all timepoints (Figure 3H). ALS plasma concentrations were approximately 5‐times lower than in the cell reservoir at the early timepoints (average D3‐14 1.25 µg mL^−1^ vs. 6.33 µg mL^−1^), while systemic exposure remained 50‐times lower, with average levels of 0.11 µg mL^−1^ in liver, 0.17 µg mL^−1^ in kidney, and 0.14 µg mL^−1^ in spleen (Figure 3I,J). However, ALS appeared to accumulate in plasma over time (3.99 ± 2.1 µg mL^−1^ by day 30), perhaps due to slow clearance [44, 45]. Anti‐CD40L showed slower release after day 3 (Figure 3K). Locally, we observed accumulation in the cell reservoir at early timepoints, particularly day 7 (35.36 ± 7.5 µg mL^−1^) (Figure 3L) while circulating levels in plasma (Figure 3M) remained at about 20‐times lower than in the proximity of the device (average 1.33 µg mL^−1^ vs. 22.13 µg mL^−1^). However, systemic distribution showed accumulation in the kidney (1.39 ± 0.23 µg mL^−1^ by day 30) (Figure 3N), possibly due to expression of CD40 in renal epithelial cells and podocytes which can be enhanced by systemic inflammation [46, 47]. The cumulative release of anti‐CD2 exhibited first‐order kinetics (Figure 3O) with local and systemic levels consistent with such a model, displaying an increase in concentration which peaked around day 7 (6.97 ± 2.02 µg mL^−1^ in the cell reservoir and 0.61 ± 0.55 µg mL^−1^ in plasma), followed by a slow decrease (Figure 3P–R). In this case, the drug concentration in plasma and systemic organs were 10‐ and 250‐times lower than the local microenvironment, respectively (average 0.38 µg mL^−1^ and 0.02 µg mL^−1^ vs. 4.93 µg mL^−1^). Finally, anti‐IL6 showed similar release dynamics to ALS and anti‐CD40L (Figure 3S) with concentrations in the cell reservoir and fibrotic capsule that were comparable and stable through day 30 (Figure 3T). Plasma levels indicated broader systemic exposure (Figure 3U), with 4‐ to 10‐ times reduced concentration compared to the local cell reservoir compartment (average 0.84 µg mL^−1^ vs. 4.73 µg mL^−1^). Concentrations in systemic organs were ∼150‐times lower (average 0.03 µg mL^−1^ vs. 4.73 µg mL^−1^), with consistent distribution across tissues (Figure 3V).

Together, these findings show that the NICHE enabled sustained in situ release of immunosuppressive agents into the local transplant microenvironment, with varying degrees of systemic exposure depending on the pharmacokinetic profile of each agent. CTLA4‐Ig, ALS and anti‐CD2 exhibited the highest local retention, whereas anti‐CD40L and anti‐IL6 displayed broader tissue distribution.

### 2.3 Characterization of Immune Response in the Absence of Immunosuppression

To characterize the baseline immune response to allogeneic islet transplantation in the subcutaneous site, we assessed local and systemic immune cell dynamics over a 30‑day period in the absence of immunosuppressive therapy. Immune cell composition at the pre‐transplant baseline (day −3, prior to islet loading) was compared with post‐transplant timepoints (days 7, 14, and 30) to define the temporal progression of immune infiltration following implantation. For subsequent treatment assessments, local immune profiling results are presented as changes relative to untreated controls, derived from cell‐type frequencies calculated within their respective parent populations. As such, these data capture compositional shifts within the immune landscape of the transplant microenvironment. Collectively, these findings provide a reference for the temporal progression of immune activation and infiltration, establishing a foundation for evaluating the effects of locally delivered immunosuppression.

**FIGURE 4:**
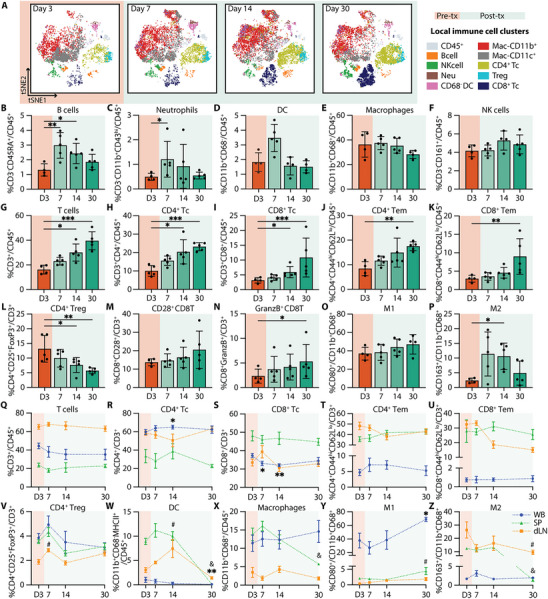
Local and systemic immune response without immunosuppression. (A) CyTOF tSNE plots of infiltrating immune cells in the NICHE cell reservoir. Day 3 shows baseline populations pre‐transplant (orange) and days 7, 14, and 30 indicate the post‐transplant period (green). CyTOF data represented as % of (B) B cells, (C) neutrophils, (D) DC, (E) macrophages, (F) NK cells, (G) T cells, (H) CD4^+^ T cells, (I) CD8^+^ T cells, (J) CD4^+^ and (K) CD8^+^ effector memory (Tem) in CD45^+^ cells; (L) CD4^+^ Treg, (M) CD28^+^CD8^+^ T cells, and (N) GranzymeB^+^CD8^+^ T cells in CD3^+^ cells; (O) M1 and (P) M2 macrophages in total macrophages (CD11b^+^CD68^+^) for the control group without immunosuppression (*n =* 4–5 per timepoint). Flow cytometry data of immune cell population trends from whole blood (WB; blue), spleen (SP; green), and draining lymph node (dLN; yellow), over a 30‐day period (*n =* 4–5 per timepoint). Data presented as % of (Q) CD3^+^ T cells in CD45^+^ cells; (R) CD4^+^ T cells, (S) CD8^+^ Tc, (T) CD4^+^ Tem, (U) CD8^+^ Tem, and (V) CD4^+^ Treg in CD3^+^ T cells; (W) DC (CD11b^+^CD68^−^MHCII^+^), and (X) macrophages in CD45^+^ cells; (Y) M1 and (Z) M2 in total macrophages. All comparisons are made with D3 baseline frequencies for local CyTOF (B‐P), WB (^*^), SP (&), and LN(#). Data shown as mean ± SEM for visualization, statistical testing was performed using Kruskal–Wallis with Dunn's post hoc test. (^*^, # or & *p* < 0.05; ^**^, ## or && *p* < 0.01; ^***^, ### or &&& *p* < 0.001).

Single‐cell mass cytometry (CyTOF) analysis of the NICHE cell reservoir revealed a dynamic pattern of immune cell infiltration during the post‐transplant period (Figure 4A). The absolute number of infiltrating CD45^+^ live cells increased post‐transplant and peaked at day 14 (day 11 post‐transplant) (Figure S3), providing a quantitative context for the relative changes observed across immune subsets. The pre‐transplant local immune infiltrate consisted primarily of CD11b^+^ and CD11c^+^ myeloid cells, including both macrophages (CD68^+^) and dendritic cells (DCs; CD11b^+^CD68^−^). Frequencies of B cells (CD3^−^CD45RA^+^) and neutrophils (CD3^−^CD43^hi^CD11b^+^) increased significantly by day 7 (day 4 post‐transplant), alongside a trend toward increased local DCs, consistent with early innate immune responses (Figure 4B–D). In contrast, the proportion of local macrophages and NK cells (CD3^−^CD161^+^) remained stable over time (Figure 4E,F).

A progressive accumulation of adaptive immune cells was evident in the post‐transplant period. Total T cells (CD45^+^CD3^+^) increased significantly by day 14 (Figure 4G), with expansion of both CD4^+^ and CD8^+^ subsets (Figure 4H,I), consistent with the overall increase in absolute infiltrating cell numbers. Notably, while the total CD45^+^ infiltrate peaked at day 14, the proportion of T cells remained significantly elevated through day 30. Effector memory phenotypes (Tem: CD44^hi^CD62L^lo^) within both subsets followed a similar pattern, increasing by day 14 and reaching significantly higher levels at day 30 (Figure 4J,K). In contrast, regulatory T cells (CD4^+^CD25^+^FoxP3^+^) declined significantly at days 14 and 30 (Figure 4L). CD28^+^ expression in CD8 Tc showed an increased trend (Figure 4M), and the proportion of granzymeB expressing CD8^+^ T cells (granzymeB^+^CD8^+^) were significantly increased at day 30 (Figure 4N), suggesting cytotoxic T cell activation. While M1‐like macrophages (CD80^+^) remained stable, M2 polarization (CD163^+^) showed a significant increase at day 14 but decrease thereafter (Figure 4O,P). Consistent with the progressive immune infiltration observed, explanted NICHE devices at day 30 showed disrupted islet morphology with immune cell infiltration and absence of insulin‐positive staining (Figure S4A,B).

Systemic immune profiling of whole blood (WB), spleen (SP), and draining lymph node (dLN) revealed complementary dynamics. Complete blood counts (CBC) showed no significant changes over time nor across groups (Figure S5). However, CD3^+^ T cell frequencies declined initially in WB and SP but recovered by day 30 (day 27 post‐transplant), while dLN showed a transient increase at day 7 (Figure 4Q). CD4 Tc significantly increased in WB at day 14, whereas CD8 Tc decreased from day 7 to 14 (Figure 4R,S). Effector memory T cells (CD4^+^ and CD8^+^ Tem) declined in dLN throughout the post‐transplant period, with minimal change in WB or SP, suggesting trafficking to the graft site (Figure 4T,U). Tregs peaked transiently at day 7, then decreased across all compartments (Figure 4V). Systemic DCs (CD11b^+^CD68^−^MHCII^+^) were significantly increased in the dLN at day 14 but declined in WB and SP by day 30 (Figure 4W). Macrophages (CD11b^+^CD68^+^) increased in SP at day 7, but significantly declined by day 30 (Figure 4X). M1‐like macrophages had a marked increase in WB and dLN by day 30 (Figure 4Y), while M2‐like macrophages declined in the dLN and SP compartments (Figure 4Z).

Collectively, these findings reveal a temporally coordinated immune response marked by an initial myeloid cell influx, followed by sustained effector T cell activation with a reduced regulatory phenotype. This progression underscores a critical window for immunomodulatory intervention within the first 10 days post‐transplant and provides a reference to assess how localized immunosuppression can reshape the transplant immune landscape.

### 2.4 Local Delivery of CTLA4‐Ig Suppresses Effector Immune Responses but Depletes Tregs

To evaluate the immunomodulatory effects of CTLA4‐Ig delivered locally in the NICHE, we compared the immune cell compositions at the transplant site and systemic compartments between treated and untreated rats over a 30‐day period. It should be noted that day 3 results represent the pre‐transplant timepoint, reflecting 3 days of localized IS release.

**FIGURE 5:**
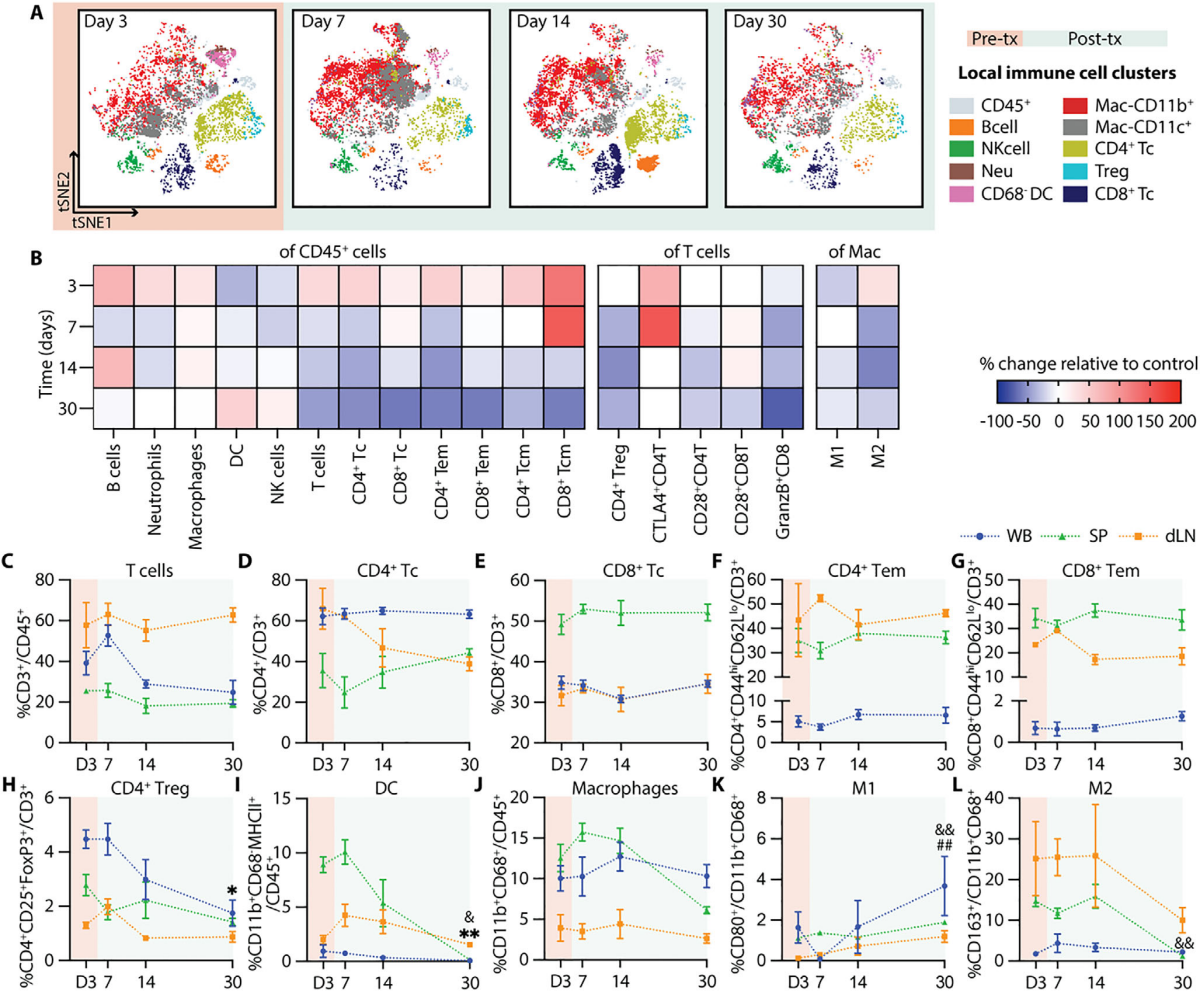
Local and systemic changes in immune cell populations with CTLA4‐Ig local delivery. (A) CyTOF tSNE plots of infiltrating immune cell populations in the NICHE cell reservoir in rats treated with locally delivered CTLA4‐Ig. Pre‐transplant (day 3) is framed in orange; post‐transplant days 7, 14, and 30 in green. (B) CyTOF analysis of NICHE cell reservoir tissue at days 3, 7, 14, and 30 post‐drug loading. All data are presented as mean % change relative to the untreated control group (*n*  =  4‐5 per timepoint/group). Descriptive statistics and statistical testing for CyTOF data are provided in the Supplementary File. Flow cytometry data of immune cell population trends from whole blood (WB; blue), spleen (SP; green), and draining lymph node (dLN; yellow), over a 30‐day period (*n =* 4–5 per timepoint). Data presented as % of (C) CD3^+^ T cells in CD45^+^ cells; (D) CD4^+^ T cells, (E) CD8^+^ Tc, (F) CD4^+^ Tem, (G) CD8^+^ Tem, and (H) CD4^+^ Treg in CD3^+^ T cells; (I) DC (CD11b^+^CD68^−^MHCII^+^), and (J) macrophages in CD45^+^ cells; (K) M1 and (L) M2 in total macrophages. Flow cytometry data comparisons are made with D3 baseline frequencies for WB (^*^), SPL (&) and LN(#). Data shown as mean ± SEM for visualization, statistical testing was performed using Kruskal–Wallis with Dunn's post hoc test. (^*^, # or & *p* < 0.05; ^**^, ## or && *p* < 0.01; ^***^, ### or &&& *p* < 0.001).

CTLA4Ig‐treated tSNE projections of the local immune landscape revealed no apparent increase in immune cell infiltration during the post‐transplant period compared to day 3, particularly in lymphoid populations at day 30 (Figure 5A). Notably, CTLA4‐Ig was the only treatment group that showed a significant reduction in the absolute number of infiltrating immune cells, observed at day 30 (Figure S3B). Quantitative heatmap analysis showed an early (day 7) reduction of B cells and innate immune subsets, including neutrophils, DCs, and NK cells (Figure 5B). Notably, B cell frequencies showed an increase by day 14 following an initial decrease at day 3, and DC frequencies trended higher by day 30 relative to both untreated controls and the CTLA4Ig‐treated day 3 pre‐transplant cohort (Supplementary File). CTLA4‐Ig also reduced local infiltration of CD4^+^ and CD8^+^ T cells, including their effector memory (Tem) and central memory (Tcm: CD44^hi^CD62L^hi^) subsets, at later post‐transplant timepoints (days 14 and 30). In line with this, CD28^+^ CD4^+^ and CD8^+^ T cells were reduced at day 30. Cytotoxic activation was also diminished across all timepoints, as evidenced by reduced granzymeB^+^CD8^+^ Tc, with a ∼75% reduction (*p* < 0.05) by day 30 (Figure 5B). Importantly, CTLA4‐Ig treatment led to a significant reduction in Tregs, a known limitation of this therapy [48], with decreases ranging ∼40%–55% across post‐transplant timepoints. Additionally, both M1‐like and M2‐like macrophages were reduced, suggesting diminished myeloid cell recruitment, impaired Tcell‐mediated polarization cues, or reduced surface detection of CD80 and CD86 due to epitope masking by CTLA4‐Ig binding [49]. Histological analysis of CTLA‐4Ig treated explanted devices at day 30, showed preserved islet structure with insulin‐positive staining (Figure S4C,D).

Systemic immune profiles showed similar directional changes to those observed locally. The proportion of T cells remained stable in SP and dLN, but showed a transient increase followed by a decline in WB (Figure 5C). CD4^+^ and CD8^+^ T cells, as well as their Tem subsets (Figure 5D–G) showed no significant changes in WB and SP, while CD4^+^ T cells showed a downward trend in the dLN reaching 38.76 ± 3.4% by day 30, compared to 65.93 ± 10.08% pre‐transplant (Figure 5D). Similar to the transplant microenvironment, Tregs were significantly reduced in blood by day 30 compared to the pre‐transplant timepoint and showed a relative decrease across all systemic compartments compared to untreated controls (Figure 5H; Figure S6). Systemic DCs and macrophage frequencies (Figure 5I,J) followed similar patterns to the control group, except that the observed increase of DCs in the dLN was absent with CTLA4‐Ig treatment. Notably, M1‐like macrophages in circulating blood were approximately 10 times lower than in the control group (Figure 5K), likely due to CD80 epitope masking. In contrast, M2‐like macrophage detection (Figure 5L) was unaffected, possibly reflecting Abatacept's higher affinity to B7‐1 (CD80) over B7‐2 (CD86) receptor [50].

Collectively, these findings demonstrate that local CTLA4‐Ig delivery via the NICHE device modulates adaptive immune responses at the graft site, with the most pronounced suppression observed at later post‐transplant timepoints. However, the associated depletion of Tregs highlights a critical limitation of CTLA4‐Ig as an IS agent used in conjunction with organ transplantation.

### 2.5 Local Delivery of ALS Selectively Depletes Effector T Cells while Preserving Regulatory Populations

**FIGURE 6:**
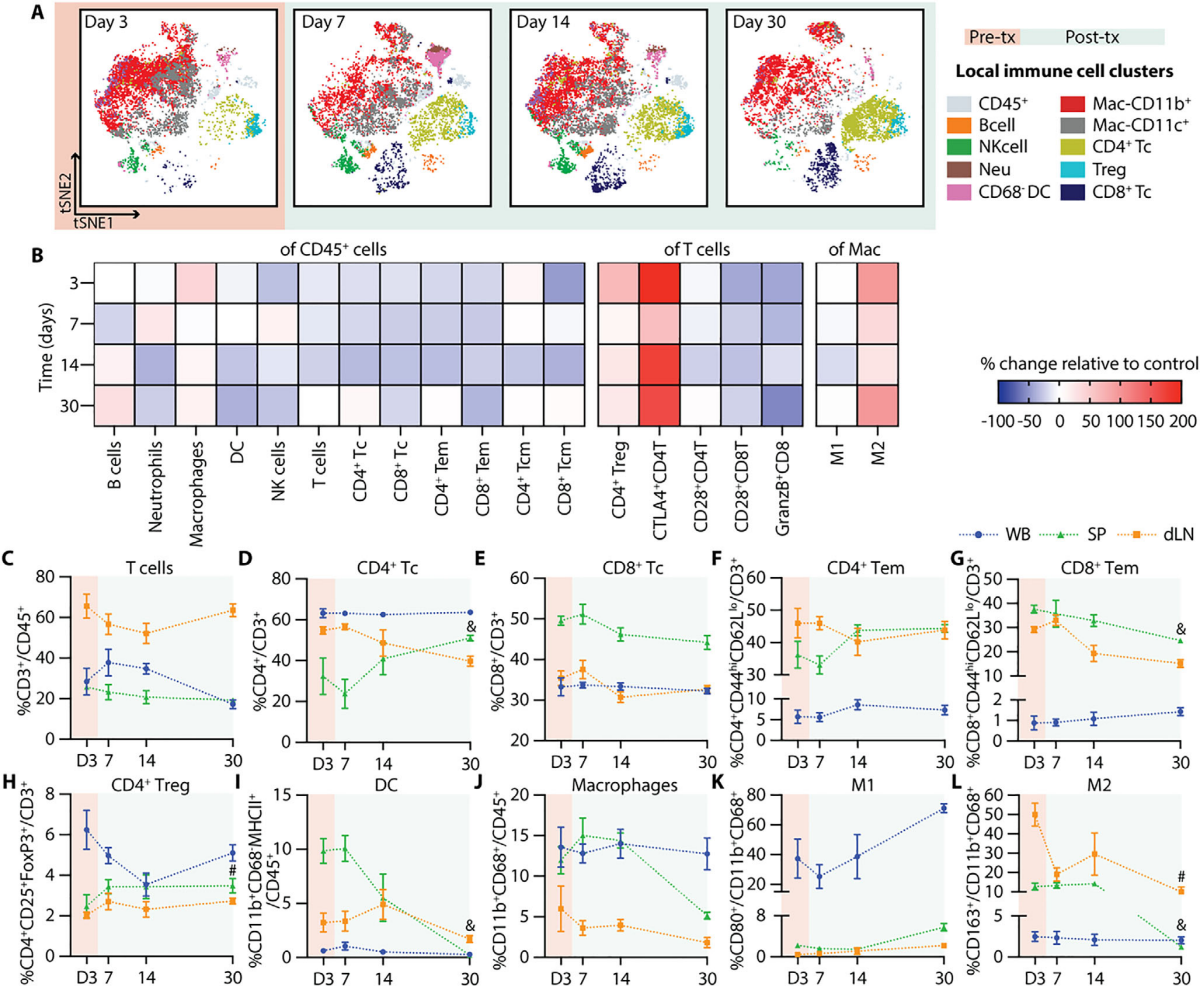
Local and systemic changes in immune cell populations with ALS local delivery. (A) CyTOF tSNE plots showing infiltrating immune cell populations in the NICHE cell reservoir over time in rats treated with locally delivered ALS. Pre‐transplant (day 3) is indicated in orange; post‐transplant days 7, 14, and 30 are shown in green. (B) CyTOF analysis of infiltrating immune cell populations in explanted NICHE devices over time (days 3, 7, 14, and 30 post‐drug loading). All data are presented as mean % change relative to the untreated control group (*n*  =  5 per timepoint/group). Descriptive statistics and statistical testing for CyTOF data are provided in Supplementary File. Flow cytometry data of immune cell population trends from whole blood (WB; blue), spleen (SP; green), and draining lymph node (dLN; yellow), over a 30‐day period (*n =* 4–5 per timepoint). Data presented as % of (C) CD3^+^ T cells in CD45^+^ cells; (D) CD4^+^ T cells, (E) CD8^+^ Tc, (F) CD4^+^ Tem, (G) CD8^+^ Tem, and (H) CD4^+^ Treg in CD3^+^ T cells; (I) DC (CD11b^+^CD68^−^MHCII^+^), and (J) macrophages in CD45^+^ cells; (K) M1 and (L) M2 in total macrophages. Comparisons are made with D3 baseline frequencies for WB (^*^), SPL (&), and LN(#). Data shown as mean ± SEM for visualization, statistical testing was performed using Kruskal–Wallis with Dunn's post hoc test. (^*^, # or & *p* < 0.05; ^**^, ## or && *p* < 0.01; ^***^, ### or &&& *p* < 0.001).

ALS is widely used clinically in organ transplantation as an induction agent for T cell depletion; however, its effects following localized administration remain poorly characterized. CyTOF analysis of the NICHE cell reservoir revealed a reduction in CD3^+^ T cells even prior to islet transplantation (day 3), with sustained suppression through day 14 (Figure 6A,B). Compared to the untreated controls, ALS treatment suppressed adaptive immune subsets, CD4^+^ Tc remained ∼16%–34% lower through day 14, while CD8^+^ T cells remained ∼16%–31% lower through day 30, with their respective Tem showing similar reductions (Figure 6B). Notably, Tregs showed a sustained longitudinal increase of ∼12%–67% locally across all timepoints. A corresponding significant increase in CTLA4^+^CD4^+^ T cells was also observed across all timepoints, potentially reflecting the enrichment of regulatory phenotypes among the residual CD4^+^ pool [41]. In contrast, CD28^+^ subsets of both CD4^+^ and CD8^+^ T cells were markedly reduced, along with granzymeB^+^ CD8^+^ T cells, indicating effective suppression of cytotoxic function. B cells were decreased by 22% at day 7 but subsequently increased relative to controls. Among innate immune subsets, DCs and NK cells were reduced through day 30. Macrophage profiling showed stable M1‐like populations, whereas M2‐like macrophages were consistently ∼26%–95% increased through day 30, suggesting a shift toward anti‐inflammatory macrophage polarization. Additionally, explanted NICHE devices at day 30 contained insulin‐positive islet grafts (Figure S4E,F).

Systemic flow cytometry showed minimal changes across WB, SP, or dLN (Figure 6C–L). A decreasing trend in circulating T cells was observed in WB, while Tregs declined at day 14 but recovered by day 30. Frequencies of DCs and total macrophages remained stable; however, the characteristic increase in DCs observed in the dLN of untreated controls at day 14 was absent following ALS treatment, and a significant reduction in splenic DCs was observed at day 30. M1‐like macrophages appeared unaffected, whereas M2‐like macrophages showed an initial decline followed by a rebound at days 14 and 30, particularly in dLN. Overall, systemic changes remained largely unaffected relative to the untreated control group (Figure S7).

In summary, ALS delivered locally via the NICHE platform effectively depleted effector T cell subsets while preserving Tregs and promoting M2‐like macrophage polarization, suggesting a favorable profile for tolerance induction.

### 2.6 Local Delivery of Anti‐CD40L Provides Selective Modulation of T Cell and Antigen‐Presenting Cell Activity

**FIGURE 7:**
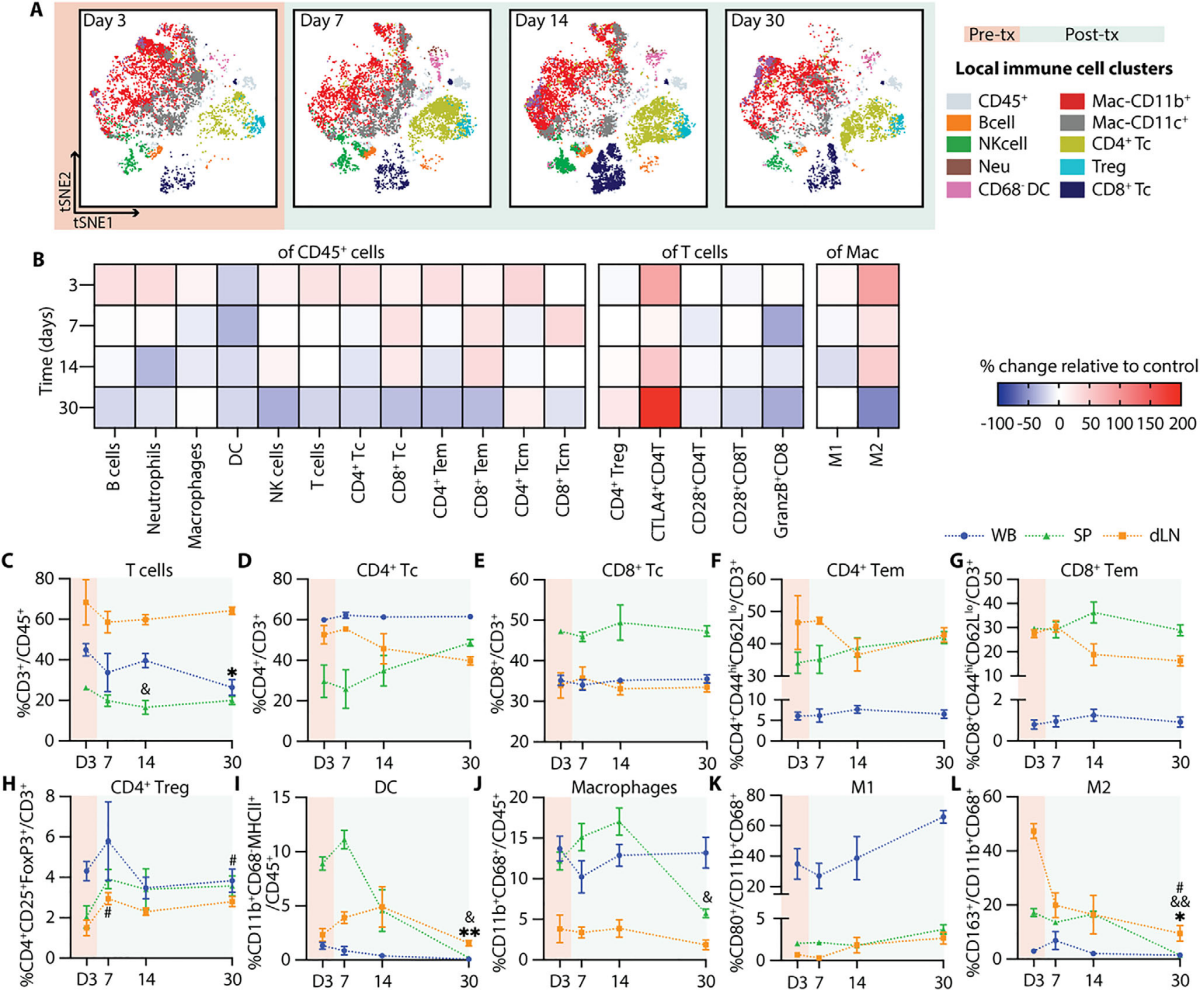
Local and systemic changes in immune cell populations with anti‐CD40L local delivery. (A) tSNE plots showing infiltrating immune cell populations in the NICHE cell reservoir over time in rats treated with locally delivered anti‐CD40L. Pre‐transplant (day 3) is indicated in orange; post‐transplant days 7, 14, and 30 are shown in green. (B) CyTOF analysis of infiltrating immune cell populations in explanted NICHE devices over time (days 3, 7, 14, and 30 post‐drug loading). All data are presented as mean % change relative to the untreated control group (*n*  =  4‐5 per timepoint/group). Descriptive statistics and statistical testing for CyTOF data are provided in Supplementary File. Flow cytometry data of immune cell population trends from whole blood (WB; blue), spleen (SP; green), and draining lymph node (dLN; yellow), over a 30‐day period (*n =* 4–5 per timepoint). Data presented as % of (C) CD3^+^ T cells in CD45^+^ cells; (D) CD4^+^ T cells, (E) CD8^+^ Tc, (F) CD4^+^ Tem, (G) CD8^+^ Tem, and (H) CD4^+^ Treg in CD3^+^ T cells; (I) DC (CD11b^+^CD68^−^MHCII^+^), and (J) macrophages in CD45^+^ cells; (K) M1 and (L) M2 in total macrophages. Comparisons are made with D3 baseline frequencies for WB (^*^), SPL (&) and LN(#). Data shown as mean ± SEM for visualization, statistical testing was performed using Kruskal–Wallis with Dunn's post hoc test. (^*^, # or & *p* < 0.05; ^**^, ## or && *p* < 0.01; ^***^, ### or &&& *p* < 0.001).

In allograft rejection, CD40‐CD40L interactions play a critical role in T cell priming, B cell activation, and dendritic cell licensing, making CD40L blockade an attractive target for localized immune modulation. CyTOF tSNE projections of CD45^+^ cells revealed a relatively stable immune landscape within the NICHE cell reservoir across the post‐transplant period (Figure 7A). Relative to untreated controls, B cells showed an early increase of 31% at day 3, followed by a 20% reduction at day 30, while DCs were reduced at days 14 and 30 (Figure 7B), consistent with inhibition of CD40L‐mediated signaling [51]. CD4^+^ and CD8^+^ T cells, including effector memory subsets declined by day 30, suggesting reduced antigen‐specific T cell activation. Regulatory T cells remained unchanged through day 14 but increased by 23% at day 30, and CD4^+^CTLA4^+^ T cells were increased across all timepoints, indicative of enhanced local immunoregulation. CD28^+^ T cells and granzymeB^+^CD8^+^ T cells were reduced, pointing at effective suppression of cytotoxic activation. Macrophage polarization was altered, with a transient increase in M2‐like macrophages through day 14 but decreasing by 58% at day 30, while M1‐like subsets remained unchanged. Consistent with this sustained immunosuppression and maintenance of regulatory populations, explanted devices at day 30 showed preserved islet architecture with a high‐density of insulin‐positive cells (Figure S4G,H).

Systemic immune profiling revealed limited effects following local anti‐CD40L treatment (Figure 7C–L). T cell subsets in SP and dLN remained stable, though a significant reduction in circulating T cells was observed in WB on day 30 compared to pre‐transplant baseline (Figure 7C). Tregs were significantly increased in the dLN at days 7 and 30 compared to pre‐transplant baseline (Figure 7H). DC frequencies in the dLN showed a small increase at day 14 relative to pre‐transplant baseline but remained below levels observed in untreated controls (Figure 7I; Figure S8C). M2‐like macrophages were reduced in dLNs by day 30 compared to pre‐transplant baseline, parallel to the decline seen locally at the same timepoint. On day 3, lymphoid populations accumulated locally (Figure 7B), coinciding with reduced cell frequencies in draining lymph nodes relative to control (Figure S8).

Together, these results show that local CD40L blockade via the NICHE device attenuated T cell co‐stimulation, cytotoxic compartments, and APC activity, while sparing regulatory cell populations.

### 2.7 Local Delivery of Anti‐CD2 and its Immunomodulatory Effect

**FIGURE 8:**
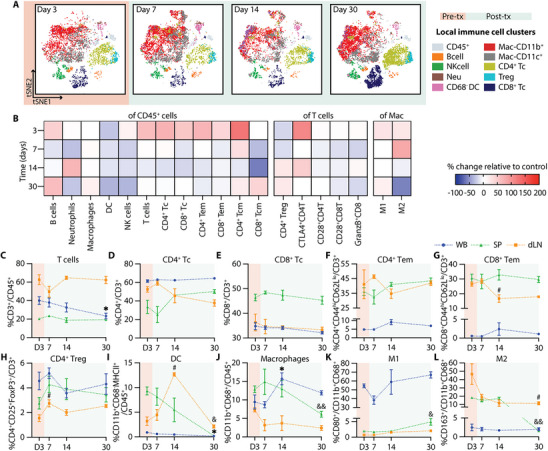
Local and systemic changes in immune cell populations with anti‐CD2 local delivery. (A) CyTOF tSNE plots showing infiltrating immune cell populations in the NICHE cell reservoir over time in rats treated with locally delivered anti‐CD2. Pre‐transplant (day 3) is indicated in orange; post‐transplant days 7, 14, and 30 are shown in green. (B) CyTOF analysis of infiltrating immune cell populations in explanted NICHE devices over time (days 3, 7, 14, and 30 post‐drug loading). All data are presented as mean % change relative to the untreated control group (*n*  =  4‐5 per timepoint/group). Descriptive statistics and statistical testing for CyTOF data are provided in Supplementary File. Flow cytometry data of immune cell population trends from whole blood (WB; blue), spleen (SP; green), and draining lymph node (dLN; yellow), over a 30‐day period (*n =* 4–5 per timepoint). Data presented as % of (C) CD3^+^ T cells in CD45^+^ cells; (D) CD4^+^ T cells, (E) CD8^+^ Tc, (F) CD4^+^ Tem, (G) CD8^+^ Tem, and (H) CD4^+^ Treg in CD3^+^ T cells; (I) DC (CD11b^+^CD68^−^MHCII^+^), and (J) macrophages in CD45^+^ cells; (K) M1 and (L) M2 in total macrophages. Comparisons are made with D3 baseline frequencies for WB (^*^), SPL (&), and LN(#). Data shown as mean ± SEM for visualization, statistical testing was performed using Kruskal–Wallis with Dunn's post hoc test. (^*^, # or & *p* < 0.05; ^**^, ## or && *p* < 0.01; ^***^, ### or &&& *p* < 0.001).

CD2 blockade was assessed in the NICHE platform during allogeneic islet transplantation to characterize its local immunomodulatory effects. CyTOF analysis of the NICHE cell reservoir revealed an immunosuppressive profile during the early (days 7–14) post‐transplant period, after an increase in T cell populations at day 3 relative to untreated controls (Figure 8A). Compared to untreated controls, anti‐CD2 treatment led to reductions in DCs and NK cells of approximately 15%–38% and 16%–36%, respectively, across post‐transplant timepoints, while CD4^+^ and CD8^+^ T cells showed only reductions of approximately 15%–20% at day 14 post‐transplant (Figure 8B). Interestingly, Treg frequencies increased by 22%–24% at days 14 and 30, suggesting enrichment of regulatory phenotypes, while CD28^+^ T cell subsets showed a 10%–15% reduction relative to control. GranzymeB^+^CD8^+^T cells were reduced by 20%–22% at days 7 and 30, suggesting partial suppression of cytotoxic function. Among myeloid populations, M2‐like macrophages were transiently elevated at day 7 (+82%) but were markedly reduced by day 30 (‐59%), while M1‐like macrophages remained stable. Consistent with these findings, explanted NICHE devices at day 30 showed preserved islet morphology and insulin‐positive staining (Figure S4I,J).

Systemic immune profiling revealed minimal perturbations across compartments (Figure 8C–L). A significant reduction in circulating T cells was observed in WB on day 30 compared to day 3 baseline (Figure 8C), which was also relatively lower than in untreated controls (Figure S9A). Systemic Treg frequencies remained largely unchanged compared to pre‐transplant baseline (Figure 8H), with only a relative increase noted in WB at day 30 compared to control (Figure S9A). Dendritic cells in the dLN increased significantly by day 14 (Figure 8I), mirroring trends in untreated controls (Figure 4W), but were significantly reduced compared to baseline at day 30 in SPL and WB. The macrophage population in WB increased significantly at day 14 (Figure 8J), while M2‐like macrophages were decreased in the dLN by day 30 (Figure 8L) compared to baseline.

In summary, local delivery of anti‐CD2 suppressed T cell responses while preserving local Tregs and inducing a transient, early post‐transplant shift toward an M2‐like macrophage phenotype.

### 2.8 Local Delivery of Anti‐IL6 Enhances Immune Activation rather than Suppressing it

**FIGURE 9:**
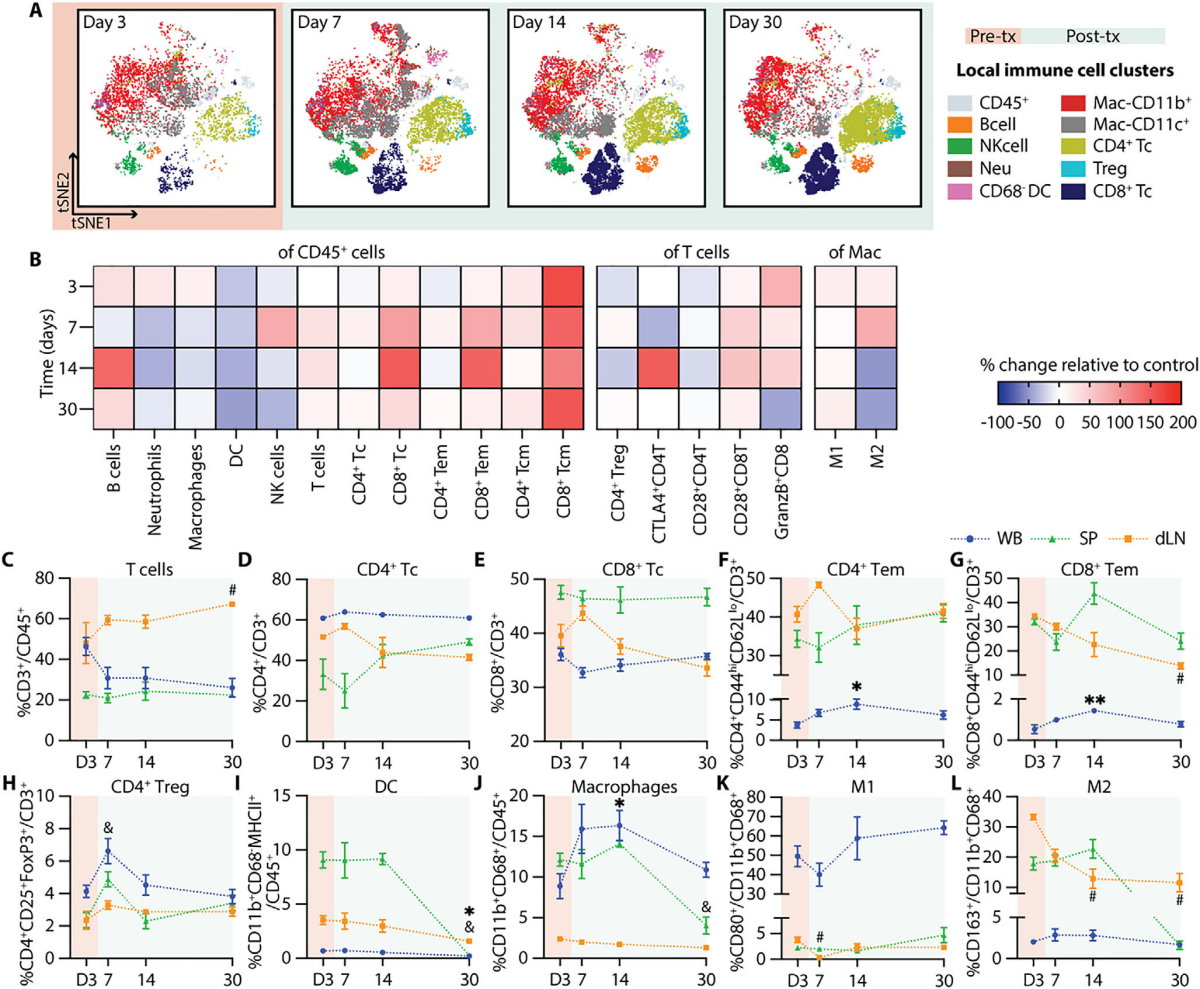
Local and systemic changes in immune cell populations with anti‐IL6 local delivery. (A) CyTOF tSNE plots showing infiltrating immune cell populations in the NICHE cell reservoir over time in rats treated with locally delivered anti‐IL6. Pre‐transplant (day 3) is indicated in orange; post‐transplant days 7, 14, and 30 are shown in green. (B) CyTOF analysis of infiltrating immune cell populations in explanted NICHE devices over time (days 3, 7, 14, and 30 post‐drug loading). All data are presented as mean % change relative to the untreated control group (*n*  =  4‐5 per timepoint/group). Descriptive statistics and statistical testing for CyTOF data are provided in Supplementary File. Flow cytometry data of immune cell population trends from whole blood (WB; blue), spleen (SP; green), and draining lymph node (dLN; yellow), over a 30‐day period (*n =* 4–5 per timepoint). Data presented as % of (C) CD3^+^ T cells in CD45^+^ cells; (D) CD4^+^ T cells, (E) CD8^+^ Tc, (F) CD4^+^ Tem, (G) CD8^+^ Tem, and (H) CD4^+^ Treg in CD3^+^ T cells; (I) DC (CD11b^+^CD68^−^MHCII^+^), and (J) macrophages in CD45^+^ cells; (K) M1 and (L) M2 in total macrophages. Comparisons are made with D3 baseline frequencies for WB (^*^), SPL (&), and LN(#). Data shown as mean ± SEM for visualization, statistical testing was performed using Kruskal–Wallis with Dunn's post hoc test. (^*^, # or & *p* < 0.05; ^**^, ## or && *p* < 0.01; ^***^, ### or &&& *p* < 0.001).

CyTOF profiling revealed increased infiltration of multiple immune populations in the cell reservoir of NICHE, releasing anti‐IL6 across the 30‐day period (Figure 9A). A broad upregulation in multiple lymphoid and myeloid populations was observed in the post‐transplant period (Figure 9B). CD4^+^ and CD8^+^ T cells, including their Tem phenotypes, were elevated and sustained through day 30. Tregs decreased by 27% on day 14. In contrast to other agents that reduced co‐stimulatory activation, anti‐IL6 led to a 44%–58% increase in CD28^+^CD8^+^ T cells at days 7–14, while granzymeB^+^CD8^+^ T cells increased by 24%–72% at days 3–14 before declining by day 30, indicating preserved or enhanced cytotoxic activity. B cells were also markedly elevated, with increased proportions of 37%–138% at days 14 and 30. Among myeloid subsets, M1‐like macrophages remained stable, while M2‐like macrophages showed a transient increase at day 7 followed by a decline thereafter. Consistent with this immune microenvironment, explanted devices at day 30 showed a resolved inflammatory response and no detectable insulin‐positive islets (Figure S4K,L). Collectively, these findings indicate that anti‐IL6 treatment did not reduce immune infiltration and was associated with a proinflammatory local immune profile.

Systemic immune profiling showed limited changes across compartments. T cells in the dLN were significantly increased by day 30 compared to pre‐transplant baseline (Figure 9C), while CD4^+^ and CD8^+^ T cells did not markedly increase over time (Figure 9D,E). In WB, CD4^+^ and CD8^+^ Tem frequencies rose at day 14 (Figure 9F,G), whereas systemic Treg frequencies remained unchanged, except for an increase in the spleen at day 7 (Figure 9H). In the dLN, DCs and total macrophages remained unchanged over time compared to baseline (Figure 9I,J) but were decreased relative to untreated controls (Figure S10C). M1‐like macrophages in the dLN were decreased at day 7 relative to both the pre‐transplant baseline and untreated controls (Figure 9K; Figure S10), whereas M2‐like macrophages were significantly reduced at days 14 and 30 compared to baseline (Figure 9L), mirroring the trend observed in the local microenvironment.

Together, these findings suggest that local IL‐6 blockade, when delivered via the NICHE device, failed to reduce immune cell infiltration and was associated with increased cytotoxic T cell activity.

## 3 Discussion

Effective immune protection of transplanted islets remains a central challenge in advancing cell‐based therapies for T1D. Although systemic immunosuppression can enable allogeneic islet engraftment, it is associated with chronic toxicity, poor patient adherence, and limited efficacy, particularly in subcutaneous transplant settings [7, 8]. In this study, we systematically assessed five immunomodulating agents with distinct mechanisms of action delivered locally via the NICHE platform: CTLA4‐Ig, ALS, anti‐CD40L, anti‐CD2, and anti‐IL6 (Figure 1). We demonstrate that sustained, local delivery of these agents enables effective immune modulation in a vascularized subcutaneous environment while significantly limiting systemic exposure. Moreover, we define safe and effective local dose ranges compatible with pancreatic islet transplantation (Figure 2) and delineate agent‐specific biodistribution profiles (Figure 3) along with their local and systemic immunomodulatory signatures.

Sustained systemic exposure to immunosuppressive agents remains the clinical standard but is associated with well‐recognized, mechanism‐based toxicities that limit dosing intensity, duration, and patient eligibility. Broad co‐stimulatory blockade, lymphocyte depletion, or cytokine inhibition at systemic levels can impair host defense, increase susceptibility to opportunistic infection and malignancy [52, 53], and disrupt immune homeostasis, thereby constraining the feasibility of prolonged or repeated administration [54]. In this context, localized drug delivery via NICHE concentrates immunosuppressive agents within the transplant microenvironment while minimizing systemic distribution. Across all agents, drug concentrations were highest within the cell reservoir, with plasma levels reduced by 5–10 fold and systemic organ exposure reduced by 100‐fold. Although demonstrated here using NICHE, these principles are likely generalizable to other local delivery strategies, including injectable long‐acting formulations or drug‐eluting biodegradable particles, underscoring the broader translational relevance of localized immunomodulation for cell transplantation.

To contextualize these findings within clinical practice, current systemic immunosuppressive regimens are defined by target plasma concentrations used to establish therapeutic efficacy. In humans or non‐human primates, systemic administration yields peak plasma concentrations generally one or two orders of magnitude higher than those observed with NICHE‐mediated local delivery in rats (Table S1) [33, 53, 54, 55, 56, 57, 58, 59, 60]. In a human setting, systemic drug levels from local NICHE delivery would be expected to be substantially lower than in rats, largely due to differences in body mass, irrespective of interspecies variation in half‐life, bioavailability, receptor expression, or clearance pathways. For example, with a comparable daily local dose of CTLA4‐Ig delivered via NICHE over 30 days, systemic concentrations in humans are estimated to be ∼70‐fold lower than in rats. This estimate assumes the same constant rate of drug input, similar subcutaneous bioavailability and clearance rates (∼0.28 and 0.91 mL kg^−1^ h^−1^ in 70 kg humans vs. 0.3 kg rats, respectively) [61, 62]. These quantitative relationships reinforce the core rationale of local immunomodulation: maximizing drug concentration at the graft site while minimizing systemic exposure to reduce the adverse effects of chronic immunosuppression.

In the local microenvironment, NICHE achieved concentrations comparable to, or exceeding, those attained with clinical systemic administration (Table S1) by tuning the concentration of the loaded immunosuppressive solution. Notably, for IgG‐based biologics such as CTLA4‐Ig, interstitial concentrations in skin and subcutaneous tissue following systemic dosing typically equilibrate to only ∼50% of plasma levels [63]. Based on this relationship, replicating the intragraft CTLA4‐Ig levels observed within NICHE would require maintaining systemic plasma concentrations nearly 20‐fold higher than those achieved with local delivery, underscoring the efficiency of this approach. Moreover, subcutaneously administered mAbs are subject to presystemic elimination via enzymatic degradation, endocytosis with lysosomal processing, FcRn‐mediated recycling, and uptake by phagocytic immune cells in draining lymph nodes, resulting in a reported bioavailability of only 52%–80% [64, 65, 66]. By circumventing these losses, NICHE delivery not only limits off‐target exposure but also amplifies therapeutic efficacy precisely where immune rejection initiates. It should also be noted that our measurements reflect the average interstitial concentrations across the entire cell reservoir. At finer spatial scales, diffusion‐driven gradients are expected to produce substantially higher concentrations at the release interface [43]. Together, these pharmacokinetic patterns highlight the relevance of considering molecular properties such as receptor affinity, molecular weight, and clearance mechanisms when selecting agents for localized immunosuppression.

While vascularization is essential for islet oxygenation and engraftment, it also provides a direct conduit for immune cell infiltration, leaving the subcutaneous site highly permissive to rejection. The untreated controls provided a clear blueprint for what must be modulated locally to delay or prevent graft rejection. In the absence of immunosuppression, the NICHE cell reservoir exhibited an early innate influx (neutrophils and dendritic cells; days 3–7) followed by progressive accumulation and activation of adaptive compartments, with sustained increases in CD4^+^ and CD8^+^ effector memory T cells, cytotoxic activation (granzymeB^+^CD8^+^), and a significant decline in Tregs at later timepoints (days 14–30). These local dynamics were accompanied by systemic changes, including an early decline of CD3^+^ T cells in WB and SP with a transient increase in dLN, consistent with trafficking toward the graft site. Together, these findings align with previously described immune rejection timelines [40] and highlight the need for temporally informed immunomodulation that addresses both early innate infiltration and later adaptive activation.

The local immunomodulatory capacity of each agent was shaped by the mechanism of action, islet compatibility, pharmacokinetics, and species specificity. Accordingly, in vitro validation was critical for confirming cross‐species reactivity and defining target dose ranges that balanced immunosuppression with islet viability (Figure 2). Interpretation of the MLR assays requires consideration of progeny‐based proliferation metrics, as lymphocyte‐depleting agents such as ALS can yield apparent increases in relative proliferation without reflecting true cell expansion. To distinguish depletion‐driven proportional effects from true proliferative responses, proliferation was interpreted alongside changes in responder cell abundance. In line with this framework, anti‐CD40L produced an increase in total NK responder abundance despite reduced CTV dilution, likely reflecting compensatory enrichment rather than increased proliferation. Here we demonstrate functional cross‐reactivity of several clinically relevant antibodies, including anti‐CD40L (MR‐1), anti‐CD2 (LoCD2bR1), and anti‐IL6 (claza), in rat immune assays, enabling their evaluation in our model. Nevertheless, the attenuated in vivo efficacy of anti‐CD2 and anti‐IL6 highlights the importance of species‐matched reagents and suggests that reduced binding affinity may partially explain attenuated or paradoxical pro‐inflammatory effects in the rat model.

CTLA4‐Ig and ALS, both previously investigated in a similar setting [37], achieved local immune modulation while maintaining confined biodistribution. CTLA4‐Ig broadly suppressed lymphocyte proliferation locally, including effector T cells and B cells, but also depleted Tregs, consistent with the non‐selective nature of CD28‐CD80/86 blockade [48]. Notably, in vitro suppression was more pronounced within the CD4^+^ and CD8^+^ T cell subsets than across the total CD3^+^ compartment, likely reflecting both the heterogeneous composition of the CD3^+^ T cell compartment and the CD28‐dependent mechanism of CTLA4‐Ig. Conventional CD4^+^ and CD8^+^ αβ T cells predominantly express CD28 and are therefore more sensitive to co‐stimulatory blockade than CD28‐low or CD28‐negative subsets, including differentiated memory T cells, double‐negative, or γδ T cells [67]. ALS, in contrast, produced marked local lymphodepletion, with up to 30% reduction in CD4^+^ and CD8^+^ T cells and a relative enrichment of Tregs, consistent with post‐depletion regulatory skewing [41]. Importantly, ALS remained safe for islets up to 50 µg mL^−1^, defining a local toxicity threshold and supporting its use as a tolerogenic induction agent without inducing systemic lymphodepletion.

Anti‐CD40L selectively attenuated effector and antigen‐presenting cell activity while preserving regulatory compartments. Although the magnitude of immune modulation observed in vivo was modest, this selective effect was sufficient to suppress rejection and maintain graft survival through day 30, suggesting that effective local CD40L blockade can achieve graft protection without broad immune depletion. Anti‐CD2 displayed limited immunomodulatory activity in vivo, despite a suppressive effect in vitro and was associated with an early increase in local lymphoid cells, likely reflecting xenogeneic responses [68]. Nevertheless, anti‐CD2 preserved Tregs and remains mechanistically attractive for combinatorial strategies targeting effector memory T cells, which are less responsive to co‐stimulatory blockade but express higher levels of CD2 [69].

In contrast to all other agents, anti‐IL6 failed to suppress immune infiltration and was associated with a net pro‐inflammatory shift, accompanied by loss of islet grafts. B cell infiltration increased by 37%–138% at later timepoints, raising concern given the role of antibody‐mediated rejection in T1D [70]. Although IL‐6 is classically pro‐inflammatory in systemic inflammatory diseases [71], its blockade in this localized setting may disrupt compensatory regulatory networks. IL‐6 signaling in CD4^+^ T cells has been shown to promote IL‐4 and suppress IFN‐γ production, favoring a Th2 polarization [72], and may indirectly sustain anti‐inflammatory myeloid phenotypes [73]. Disruption of these regulatory pathways could tilt the local immune microenvironment toward a Th1‐skewed, rejection‐prone state [74]. These findings emphasize the complexity of targeting pleiotropic cytokines in localize settings and the importance of mechanistic validation when designing local immunomodulatory regimens.

The agent‐specific immune fingerprints defined here provide a framework for rational combinatorial design for localized immunomodulation. ALS exhibited a favorable tolerogenic profile characterized by suppression of effector compartments alongside preservation or enrichment of Tregs and anti‐inflammatory myeloid polarization. Anti‐CD40L showed a complementary pattern by attenuating co‐stimulatory signaling and antigen‐presenting activity while sparing regulatory subsets, supporting its role as a local co‐stimulation backbone capable of reducing T cell priming without collapsing regulatory compartments [75]. In contrast, while CTLA4‐Ig broadly suppressed lymphoid activation, it also depleted Tregs, raising concerns regarding its use as local monotherapy when a durable regulatory balance is desired. Nonetheless, CTLA4‐Ig may retain utility in combination regiments when paired with Treg‐preserving agents and optimized for dose and exposure.

Anti‐CD2 represents a mechanistically distinct adjunct for addressing the late emergence of effector memory and cytotoxic programs observed in untreated controls, given the relative resistance of memory T cells to co‐stimulation blockade as CD28 expression declines with repeated antigen exposure [76, 77]. Although attenuated in this rat model, its mechanistic niche remains relevant for strategies aimed at improving durability through concurrent targeting of naïve and memory compartments. By contrast, IL‐6 blockade alone appeared insufficient to reprogram the graft‐site immune milieu, suggesting that cytokine modulation may require careful pairing with complementary pathways before inclusion in local regimens.

Together, these data support combinatorial strategies built around a transient, induction‐style local regimen that blunts early immune infiltration and priming at the graft site, followed by adjunct agents to reinforce control of effector memory and cytotoxic pathways that emerge later in the rejection trajectory. For example, a locally delivered combination of ALS and anti‐CD40L could provide an effective induction framework by suppressing early effector activation while preserving regulatory compartments, with the addition of anti‐CD2 to strengthen coverage of memory and cytotoxic programs that are relatively resistant to co‐stimulatory blockade. Such approaches should be designed to reshape the graft‐site immune landscape while maintaining the key advantage of high intra‐graft exposure with minimal systemic dissemination.

Some limitations should be considered. The use of non‐species‐matched antibodies constrained the assessment of some immunomodulatory effects, and the rat model lacks autoimmune components central to type 1 diabetes. Notably, no preclinical model fully recapitulates the complexity of the clinical transplant setting, and even non‐human primate studies remain limited by antibody specificity as well as species‐specific pharmacokinetics. Although biophysical factors may influence release kinetics and biodistribution, a systematic evaluation of agent‐specific transport mechanisms was beyond the scope of this study. In prior work, molecular size was identified as a dominant determinant of transport behavior [38, 78]; however, the agents evaluated here are largely IgG‐based biologics with comparable molecular weights and hydrodynamic radii. As a result, size‐dependent differences were minimized, limiting our ability to resolve more subtle biophysical interactions within the current experimental framework. While CyTOF and flow cytometry enabled detailed characterization of graft‐site immune responses, donor‐specific functional tolerance was not assessed. In addition, although biodistribution and immune profiling were comprehensive, the study was limited to a 30‐day observation period, and graft function in diabetic models was not evaluated. These choices were intentional to isolate immune modulation following localized delivery while minimizing metabolic confounders. Notably, prior studies using localized delivery of CTLA4‐Ig and ALS sustained islet function for over 150 days [37], supporting the premise that local immunomodulation does not inherently compromise graft viability. Dedicated functional studies in diabetic models will be required to extend these findings to additional agents.

## 4 Conclusion

In summary, this work establishes a comparative framework for evaluating localized immunosuppression in vascularized subcutaneous platforms for islet transplantation. NICHE provides an adaptable platform to dissect immune dynamics and guide rational design of localized regimens with minimized systemic spillover. While demonstrated here using NICHE, these principles are likely applicable to other local delivery systems. By defining agent‐specific immune profiles and highlighting combinatorial opportunities, this study lays a foundation for shifting immunosuppression from systemic to localized strategies, directly addressing a major barrier to clinical translation. Ultimately, localized immunomodulation has the potential to reduce treatment toxicity, improve adherence, and enhance quality of life for transplant patients.

## 5 Experimental Section/Methods

### 5.1 Immunosuppressants

The following immunosuppressive agents were used in this study: CTLA4‐Ig (abatacept, Orencia, Bristol‐Myers Squibb); anti‐lymphocyte serum (ALS, rabbit anti‐lymphocyte, Accurate Chemical, AIA5940/20); InVivoPlus anti‐mouse CD40L (MR‐1) (BioXCell, BP0017‐1‐R); Anti‐CD2 [LoCD2bR1] (NHPRR, AB_2819335); and anti‐IL6 [claza] (NHPRR, AB_2895638). All lyophilized agents were solubilized in sterile water for injection at the appropriate working concentrations and kept at 4°C until used in vitro or loaded into the drug reservoir of implanted NICHE devices via a transcutaneous injection procedure.

### 5.2 Cell Trace Violet (CTV) Allogeneic Mixed Lymphocyte Culture

To assess the immunosuppressive activity of each agent in vitro, MLRs were performed using CellTrace Violet‐labeled responder (recipient) and irradiated stimulator (donor) rat splenocytes. Briefly, responder splenocytes were isolated from Fisher344 rats and labeled with CellTrace Violet (Thermo Fisher Scientific, C34557) according to the manufacturer's instructions. Labeled cells were co‐cultured with irradiated (3000 cGy) Lewis rat splenocytes at a 2:1 ratio (2 × 10^5^ cells/well) in a 96‐well plate and with complete RPMI medium (200 µl). The culture medium was prepared as previously published [29], consisting of RPMI 1640 + GlutaMAX (Thermo Fisher Scientific, 61870036) supplemented with 15% heat‐inactivated human AB serum (Valley Biomedical, HP1022), 1% penicillin‐streptomycin (Thermo Fisher Scientific, 15140‐ 122), 1% non‐essential amino acids (11140‐050), 1% sodium pyruvate (11360‐070), 1% vitamins (11120‐ 052), and 1% HEPES (Corning, 25‐060‐CI). Technical triplicates were set up for each drug concentration tested. Negative controls for each drug concentration included responder cells cultured alone in MLR medium. The effect of different concentrations (0, 10, 50, and 100 µg mL^−1^) of CTLA4‐Ig, ALS, anti‐CD40L, anti‐CD2, and anti‐IL6 were independently tested in the MLR to determine the minimal effective concentrations. Cultures were incubated at 37°C and 5% CO_2_ for 4 days, followed by staining with antibodies for CD45‐FITC, CD3‐BV605, CD4‐BV510, CD8a‐PerCP, CD45RA‐PE, CD161‐RB780, CD25‐BV421, and FoxP3‐APC. Viability was assessed using Live/Dead Fixable Near‐IR Dead Cell Stain (Thermo Fisher Scientific). All antibodies are included in Table S2. Flow cytometry was performed using a Cytek Aurora cytometer, and data were analyzed using Kaluza software (version 2.1, Beckman Coulter). Proliferation was quantified as the percentage of divided cells (“CTV‐diluted”, dark blue box in Figure S1) within the remaining CTV‐labeled responder population (yellow box in Figure S1), which includes both undivided “CTV‐bright” and “CTV‐diluted” cells. Values were normalized by subtracting background proliferation in their respective negative controls. To further distinguish true proliferative responses from depletion‐driven relative effects, changes in total responder cell abundance (CTV‐bright and CTV‐diluted cells) are also included as blue‐red gradient for interpretation of depletory mechanisms. Data is presented as fold change relative to untreated (0 µg mL^−1^) groups for each IS agent tested. Population gating is shown in Figure S1.

### 5.3 Dynamic Glucose‐Stimulated Insulin Secretion (GSIS) and DNA Quantification

Islets were isolated and purified using a standard technique, as previously described [79]. Islet function following 5‐day drug exposure was assessed via perifusion (dynamic) assays to evaluate insulin secretory kinetics under glucose stimulation. The assay was performed using a PER14‐115 Perifusion system (Biorep Technologies, Miami, FL) as previously described [80]. Briefly, 100 islets per replicate were loaded in each microcolumn containing P‐4 gel matrix (Bio‐Rad Laboratories, Hercules, CA) and then perifused at 37°C with Krebs‐Ringer buffer containing sequential glucose and KCl stimuli at a flow rate of 100 µL min^−1^. Glucose stimulation for rodent islets followed the sequence: low glucose (2.8 mm) for 1 h (preconditioning), then low for 5 min, high glucose (16.7 mm) for 10 min, low for 15 min, then KCl (25 mm) for 5 min, and a final low glucose for 5 min. Effluent fractions were collected every minute and cooled to 4°C for insulin quantification by ELISA (Rat Insulin ELISA kit; Mercodia). Following perifusion, islets and surrounding slurry in each microcolumn were collected in T‐PER Tissue Protein Extraction Reagent (Thermo Fisher Scientific), vortexed, and frozen. Total DNA content was quantified using the Quant‐iT PicoGreen dsDNA Assay Kit (Thermo Fisher Scientific) to normalize insulin output per islet DNA content for each microcolumn.

### 5.4 NICHE Fabrication

The NICHE device was constructed following previously established protocols [36, 37, 38, 39, 40]. In summary, two nanoporous polyethersulfone (PES) membranes (30 nm pore size, Sterlitech) and two pairs of nylon mesh sheets (Elko Filtering) were attached to a 3D‐printed polyamide frame (dimensions: 30.4 mm × 15.4 mm × 3.8 mm) using a medical‐grade silicone adhesive (MED3‐4213, Nusil). This silicone adhesive was also utilized in forming the ports used for transcutaneous loading of immunosuppressants and islets. Prior to assembly, all components underwent autoclave sterilization. Final assembly was conducted under sterile conditions within a laminar flow cabinet. Completed NICHE devices were then subjected to ethylene oxide gas sterilization at the Current Good Manufacturing Practice (cGMP) facility of Houston Methodist Research Institute (HMRI).

### 5.5 Animal Models

Male Fischer (CDF) rats (8 weeks old; Charles River, Strain Code 002, MHC Haplotype RT1lv) were used for this study. Animals were housed in pairs under standard light/dark cycles in the Comparative Medicine Program facility at Houston Methodist Research Institute, with ad libitum access to water and a standard protein‐enriched rodent diet (Teklad Global 18%, Envigo). At designated study endpoints, animals were euthanized using isoflurane overdose, followed by confirmation of death. All procedures were performed in accordance with institutional and federal animal welfare guidelines and were approved by the Houston Methodist IACUC (Protocol #IS00007362).

### 5.6 In Vivo Study Design

NICHE devices were subcutaneously implanted as previously described [37, 40]. Prior to implantation, bone marrow‐derived MSCs from F344 rats (Cyagen) were suspended in 20% Pluronic F‐127 hydrogel prepared in DMEM (Sigma) and loaded into the cell reservoir at a density of 5 × 10^5^ MSCs per device. Devices were then surgically implanted into a subcutaneous pocket created in the rat dorsum, as previously described. Following implantation, animals were allowed to undergo a five‐week prevascularization period. After this vascularization phase, rats were randomized into treatment groups corresponding to each immunosuppressive drug: CTLA4Ig, anti‐lymphocyte serum (ALS; rat analog of ATG), anti‐CD40L, anti‐CD2, and anti‐IL6, or vehicle control (PBS). Each group consisted of 20 animals, with n = 5 rats allocated per timepoint (days 3, 7, 14, and 30 post‐drug loading). Drug solutions were loaded into the NICHE drug reservoir using sterile technique via the designated silicone ports. On day 3 post‐drug loading, a subset of animals (n = 5 per group) was euthanized to evaluate baseline immune responses and drug distribution prior to islet transplantation. The remaining animals received a subtherapeutic dose of allogeneic Lewis rat pancreatic islets (6000 IEQ kg^−1^) delivered directly into the NICHE cell reservoir to assess drug efficacy in modulating transplant immunity. Animals were euthanized at days 7, 14, and 30, and samples were collected for drug quantification. Harvested tissues included blood (via cardiac puncture), NICHE device components (cell reservoir tissue, fibrotic capsule, and overlying skin), contralateral axillary lymph node, liver, kidney, and spleen. Samples were processed for drug quantification as described in subsequent sections.

### 5.7 Ex‐Vivo Drug Quantification

Ex‐vivo drug quantification was performed as previously described [37, 40]. Briefly, harvested tissue samples were homogenized in T‐PER tissue protein extraction buffer (Thermo Scientific) supplemented with protease inhibitor cocktail (Thermo Scientific) to preserve protein integrity. Tissue homogenates and blood samples were centrifuged to remove debris and isolate plasma, then clarified supernatants were stored at −80°C until analysis. Quantification of drug concentrations in plasma and tissue homogenates was performed using immunoassay kits selected for compatibility with each therapeutic agent. CTLA4‐Ig levels were measured using a human CTLA4 ELISA (Invitrogen, BMS276TEN). ALS was detected using the HTRF Rabbit Fc kit (Revvity, 6FRIGPEG). Anti‐CD40L was quantified using a Hamster Armenian IgG ELISA Kit (Innovative Research, IHMARIGGKT), while anti‐CD2 and anti‐IL6 were quantified using a Monkey (Rhesus) Total IgG ELISA Kit (Innovative Research, IMNRSIGGKTT). All assays were conducted according to manufacturers' instructions. Fluorescence intensity was measured using a Synergy H4 multimode plate reader (BioTek), and sample concentrations were calculated based on standard curves. All samples were assayed in duplicates and averaged for analysis.

### 5.8 Immunomodulation Assessment

To assess the immunomodulatory effects of locally delivered IS agents via the NICHE device, we profiled immune cell dynamics in the transplant microenvironment and systemic compartments over the course of 30 days. At each timepoint (days 3, 7, 14, and 30 post‐drug loading), n = 5 rats/group were euthanized, and samples were collected for immune profiling. Peripheral blood, draining axillary lymph nodes, and spleens were harvested and processed into single‐cell suspensions for flow cytometry analysis. For local immune profiling, NICHE cell reservoir tissues were dissected from retrieved devices, mechanically dissociated, and enzymatically digested into single‐cell suspensions and processed for CyTOF.

### 5.9 CyTOF Analysis

Single‐cell suspensions from NICHE cell reservoir tissues were prepared as previously described [40], using enzymatic digestion with collagenase/hyaluronidase (StemCell Technologies) and DNAse I (Roche, 100 µg mL^−1^) in RPMI‐1640 medium. Following digestion, red blood cells were lysed using ACK lysis buffer, and the resulting cell suspensions were washed and filtered through 40 µm strainers to remove debris. Cells were incubated with a metal‐conjugated viability dye (Standard BioTools) for 5 min at room temperature, then washed with cell staining buffer. Surface and intracellular staining was performed using a metal‐tagged antibodies for surface and intracellular markers listed in Table S3. After staining, cells were incubated overnight at 4 °C in Cell‐ID Intercalator Ir (Standard BioTools) for DNA labeling. The next day, samples were washed, filtered, and acquired using the Helios instrument (Standard BioTools). Data were exported as FCS files and uploaded to Cytobank for preprocessing and analysis. Immune populations were annotated based on expression profiles and marker‐defined gating (Table S4). Cell population frequencies were quantified for each treatment group and timepoint. Fold changes in relative abundance compared to the untreated control group were visualized as heatmaps generated for each treatment group.

For statistical analysis, descriptive statistics are reported as median and interquartile range (IQR) in the Supplementary File. For each immune cell population within each treatment group, pre‐specified comparisons assessing time‐dependent changes relative to baseline and differences between treatment and control groups at matched timepoints were performed using a non‐parametric Kruskall‐Wallis framework followed by Dunn's post hoc test. *P* values across these seven comparisons were adjusted using the Holm method to control the family‐wise error rate within each immune population.

### 5.10 Ex‐Vivo Flow Cytometry Analysis

Single cell suspensions were prepared from harvested lymph nodes and spleen by mechanical filtration through 40 µm cell strainers in RPMI‐1640 medium (Gibco) as previously described [40]. Spleens were subjected to ACK lysis (Quality Biological) to remove red blood cells. Cells were then washed with PBS supplemented with 2% fetal bovine serum (FBS), pelleted, and resuspended in PBS for subsequent staining. Whole blood was stained without any prior washes. Cells were incubated with Fc receptor blocking antibody (anti‐CD32, BD Biosciences) for 15 min at 4°C to reduce nonspecific binding. Surface staining was performed using two predefined antibody panels (Table S5), a lymphoid panel (CD45, CD3, CD4, CD8a, CD25, and viability dye); and a myeloid panel (CD45, CD11b/c, MHC‐II, CD80, CD163, and viability dye). Cells were stained for 30 min at 4°C in the dark. After surface staining, cells from lymph nodes and spleen were fixed with Fixation/Permeabilization buffer (eBioscience, Invitrogen) for 20 min at room temperature. Whole blood cells were fixed with 1‐step Fix/Lyse Solution (eBioscience, Invitrogen). Fixed cells were then washed 1X permeabilization buffer (Invitrogen) and stained intracellularly with Foxp3 (lymphoid panel) or CD68 (myeloid panel) at 4°C for 30 min. Fluorescence‐minus‐one (FMO) and unstained controls were included for gating strategy validation. Data were acquired using an A5SE flow cytometer (BD Biosciences) and analyzed using FlowJo v10 software (FlowJo LLC). Debris, doublets, and dead cells were excluded from analysis. Lymphoid and myeloid subsets were gated on live CD45^+^ leukocytes, and immune populations were quantified as a percentage of total live CD45^+^ cells or as absolute counts where applicable. Gating strategy is exemplified in Figures S11 andS12 for lymphoid and myeloid panels, respectively.

For visualization purposes, data is shown as mean ± SEM and within each cell type and treatment group, cell frequencies during the post‐transplant period (days 7, 14, and 30) were compared with day 3 using Kruskall–Wallis with Dunn's post hoc test.

### 5.11 Islet Engraftment

NICHE devices explanted at day 30 were fixed in 10% formalin for 3 days and processed for histology analysis as previously described [40]. Tissue sections were stained with hematoxylin‐eosin (H&E) and Insulin (2D11‐H5, sc‐8033, Santa Cruz, 1:100) at the Baylor College of Medicine Pathology Core. Slides were imaged with a Keyence BZ‐X800 Microscope (Keyence) with 40x objectives.

### 5.12 Statistical Analysis

Depending on the data set, comparisons were made across timepoints to determine time‐dependent changes relative to day 3, and between treatment and control groups to determine the impact of each drug. All statistical analyses were performed using Prism 10.3.2 (Graphpad). The data is presented as mean ± standard deviation (^*^ *p* < 0.05, ^**^ *p* < 0.01, ^***^ *p* <0.001, ^****^ *p* <0.0001). CyTOF statistical analyses were performed in R (version 4.5.2), using the rstatix package for non‐parametric testing and tidyverse packages for data processing. Specific analysis method, number of replicates, and *p* values are specified in each legend.

## Author Contributions

Conceptualization is carried out by J.N.C.‐C., S.C., J.P.‐M., and A.G. Formal analysis is performed by J.N.C.‐C., S.C., M.A.W., D.M.B., A.L.A.‐G., G.E.R., R.O., H.J.S., and J.Z. Funding acquisition is secured by N.S.K. and A.G. Investigation is conducted by J.N.C.‐C., S.C., M.A.W., A.R., D.M.B., A.L.J., T.B., L.F., M.C., A.L.A.‐G., G.E.R., H.J.S., and C.A.C. Methodology is developed by J.N.C.‐C., S.C., M.A.W., J.P.‐M., C.Y.X.C., J.Z., and N.S.K. Project administration is managed by J.N.C.‐C., S.C., and N.S.K., while supervision is provided by S.H.C., N.S.K., and A.G. Visualization is carried out by J.N.C.‐C., S.C., and J.Z. The original draft is written by J.N.C.‐C. and S.C., and the manuscript is reviewed and edited by J.P.‐M., A.G., C.Y.X.C., M.W., and N.K.

## Funding

This work was supported by Breakthrough T1D (BT1D 2‐SRA‐2022‐1224‐S‐B, AG, NK), NIH NIDDK (R01 DK133610‐01, AG, NK), and Houston Methodist Research Institute (AG).

## Conflicts of Interest

The authors S.C., J.P.‐M., C.Y.X.C., and A.G. are inventors of intellectual property licensed by Continuity Biosciences. A.G. is a co‐founder and scientific advisor of Continuity Biosciences. All other authors declare they have no competing interests.

## Supporting information




**Supporting File 1**: advs74709‐sup‐0001‐SuppMat.docx.

**Supporting file 2**: advs74709‐sup‐0002‐Data.zip.

## Data Availability

The data that support the findings of this study are available from the corresponding author upon reasonable request.
