# Supplementary material for: Profiling Localized Immunomodulation and Drug Biodistribution within a Subcutaneous Vascularized Niche for Cell Transplantation
Source: Adv Sci (Weinh). 2026 Mar 6;13(28):e20914. doi: 10.1002/advs.202520914 (PMC13185831; doi:10.1002/advs.202520914)
Supplement: Supplementary file 1 — Supporting File 1: advs74709‐sup‐0001‐SuppMat.docx. [file ADVS-13-e20914-s002.docx]

Supporting Information

**Profiling localized immunomodulation and drug biodistribution within a subcutaneous vascularized niche for cell transplantation**

Jocelyn Nikita Campa-Carranza, Simone Capuani, Melissa A. Willman, Alexander Rabassa, Ashley L. Joubert, Tommaso Bo, Letizia Franco, Marzia Conte, Ana L. Anaya-García, Gabrielle E. Rome, Rim Ouni, Henry J. Seaborne, Camden A. Caffey, Dora M. Berman, Junjun Zheng, Jesus Paez-Mayorga, Corrine Ying Xuan Chua, Shu Hsia Chen, Norma S. Kenyon, Alessandro Grattoni*


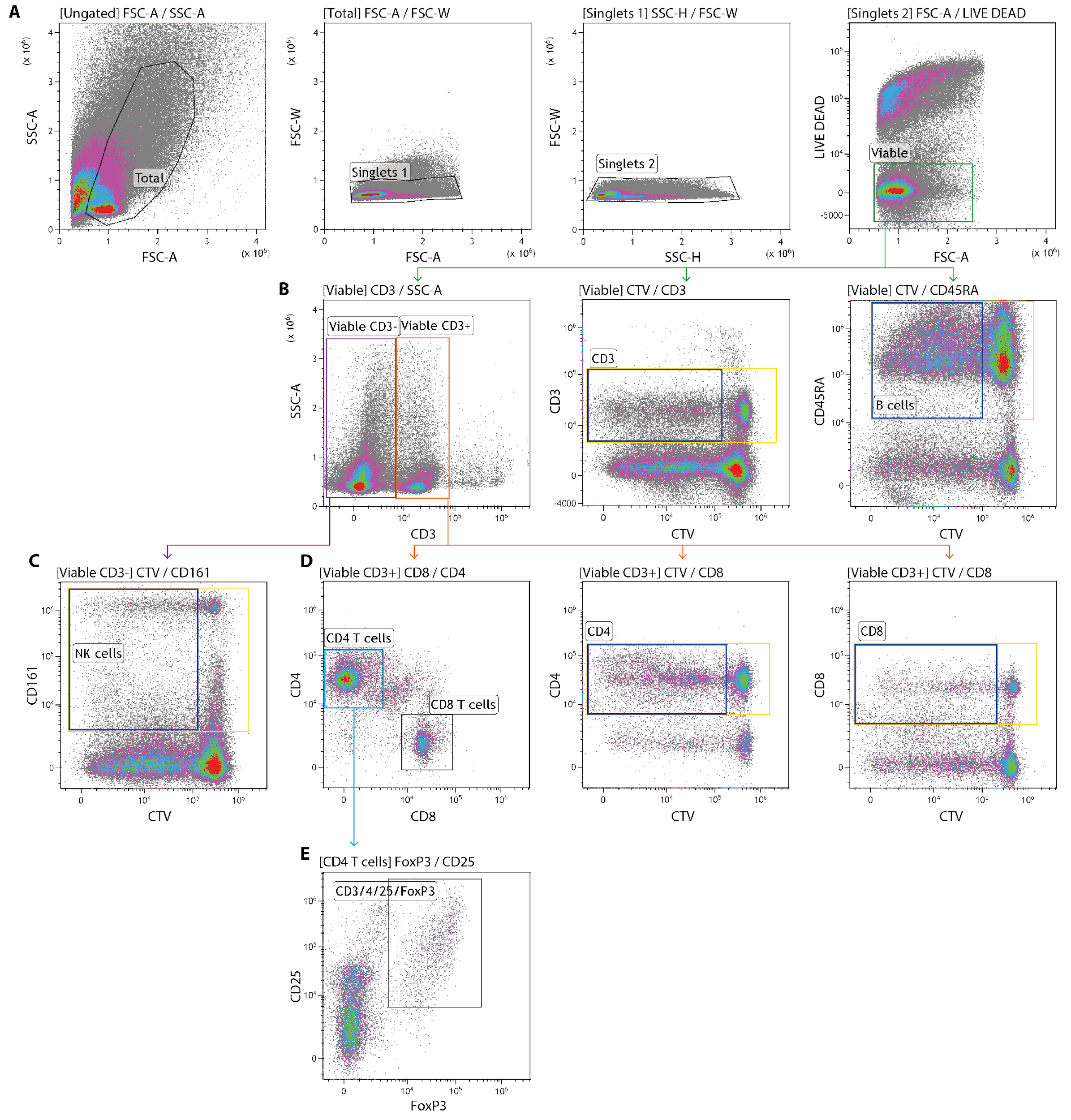


**Figure S1.** Gating strategy for rat allogeneic mixed lymphocyte reaction (MLR). Recipient (F344) rat splenocytes were isolated and labeled with cell trace violet (CTV) and cultured in media alone or with allogeneic (Lewis) rat splenocytes for 4 days, followed by flow cytometry analysis of total CTV-labeled cells and proliferating cells based on CTV dilution. Gating strategy for (A) viable cells; (B) CD3- and CD3+ cells, proliferating CD3+ cells, and proliferating B cells out of viable cells; (C) NK cells out of CD3- cells; (D) CD4+ and CD8+ T cells, proliferating CD4+, and proliferating CD8+ out of CD3+ cells; and (E) CD4+ Treg out of CD4+ T cells. The % of proliferating cells (dark blue box) was calculated from total CTV-labeled cells (yellow box).


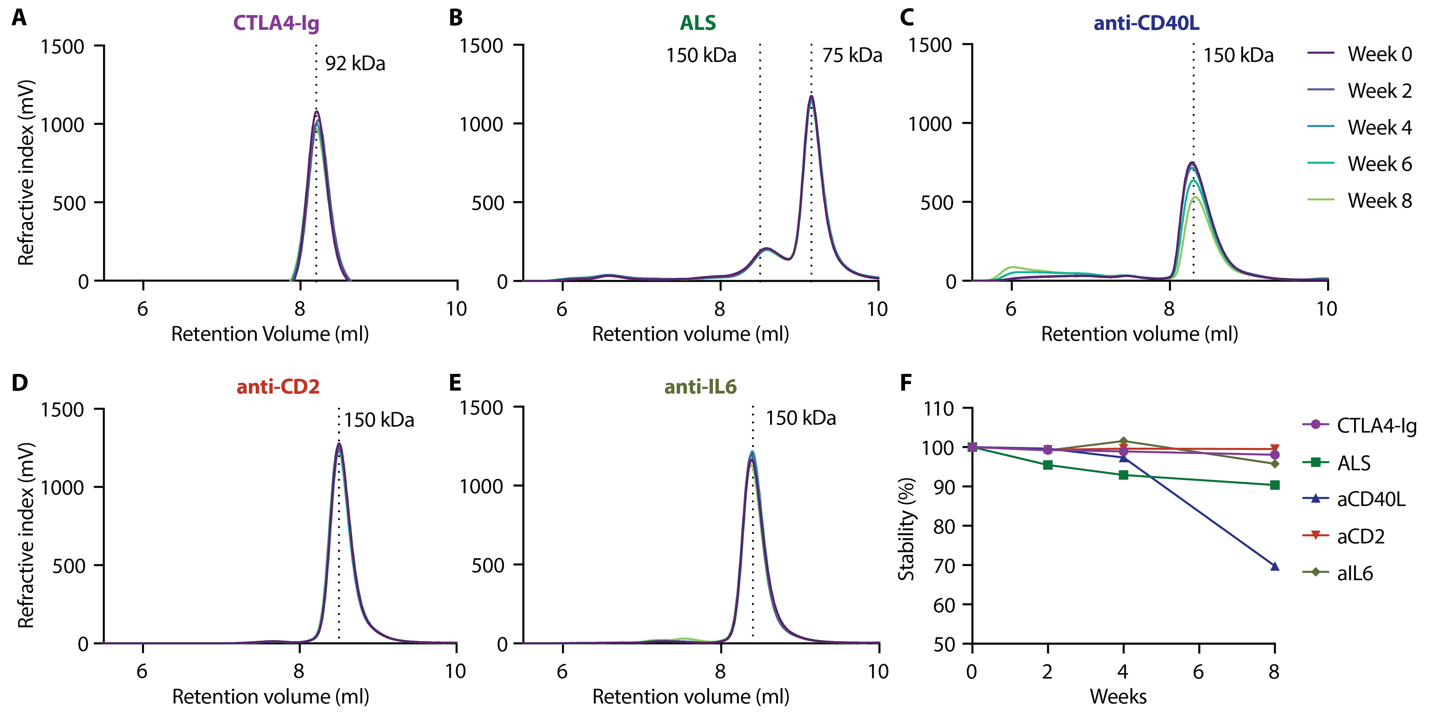


**Figure S2.** Stability of IS drugs by GPC. Gel Permeation Chromatography (GPC) was used to assess stability of (**A**) CTLA4-Ig, (**B**) ALS, (**C**) anti-CD40L, (**D**) anti-CD2, and (**E**) anti-IL6. Fresh stock (week 0) was compared with drug solutions incubated at 37°C in hermetically sealed glass vials under constant magnetic stirring. Samples were collected every 2 weeks and analyzed by GPC against fresh controls.


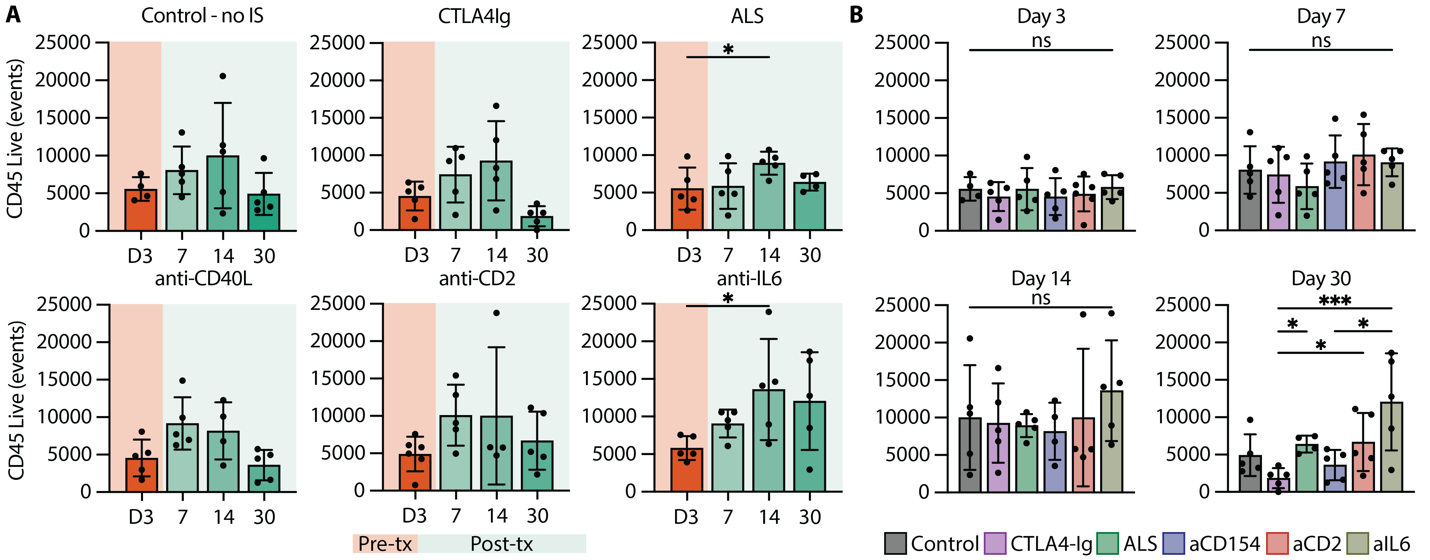


**Figure S3.** Absolute number of intra-graft infiltrating immune cells. Quantification of total number of CD45^+^ live cells included in CyTOF analysis represented as (**A**) changes over time within each group and (**B**) differences between groups at each timepoint. Comparisons are made with D3 baseline frequencies for (A) and across groups for (B). n= 4-5/timepoint/group. Mean ± SD, Kruskal-Wallis test, followed by Dunn’s post hoc test. (* p < 0.05; ** p < 0.01; *** p < 0.001).


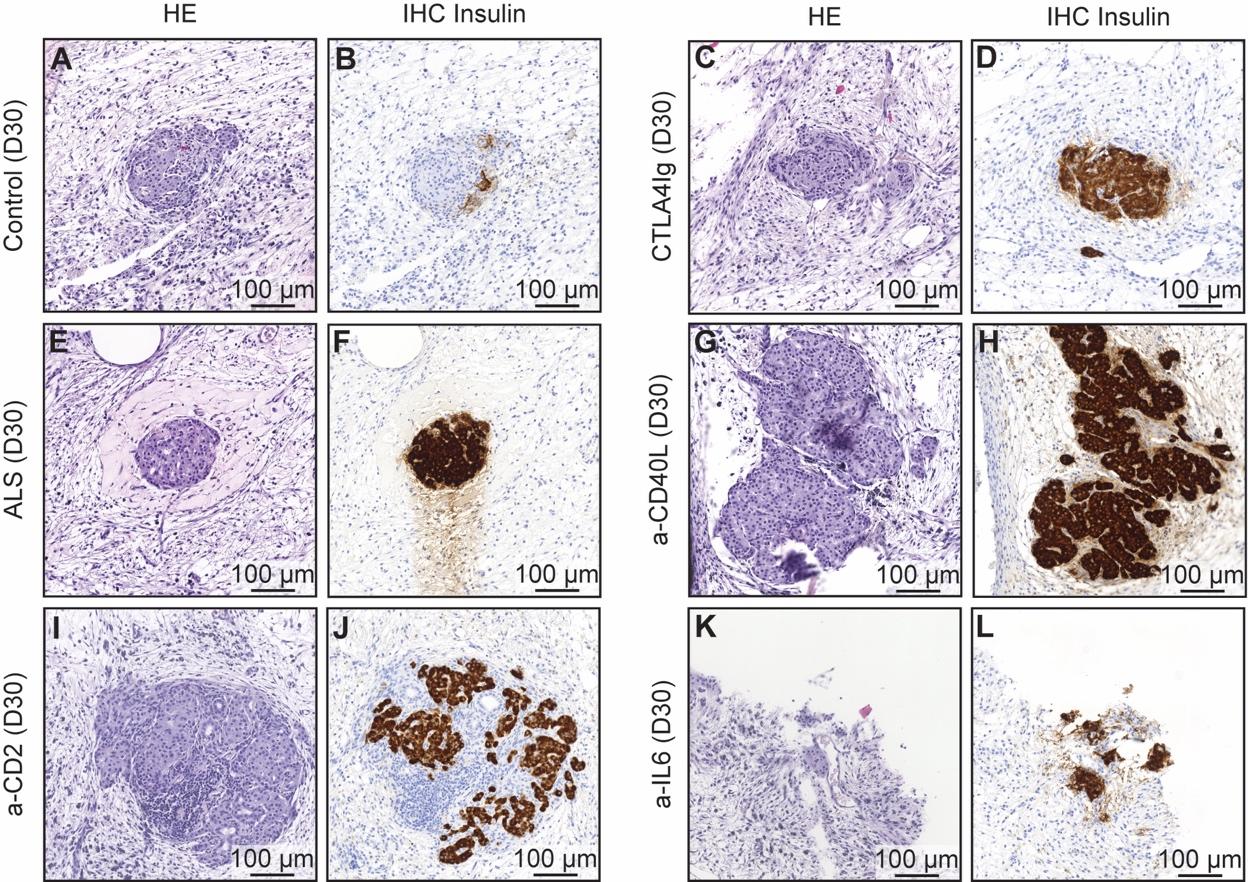


**Figure S4.** Histological assessment of islet graft survival at day 30. Representative H&E staining (left panels) and insulin immunohistochemistry (right panels) of explanted NICHE devices at day 30.


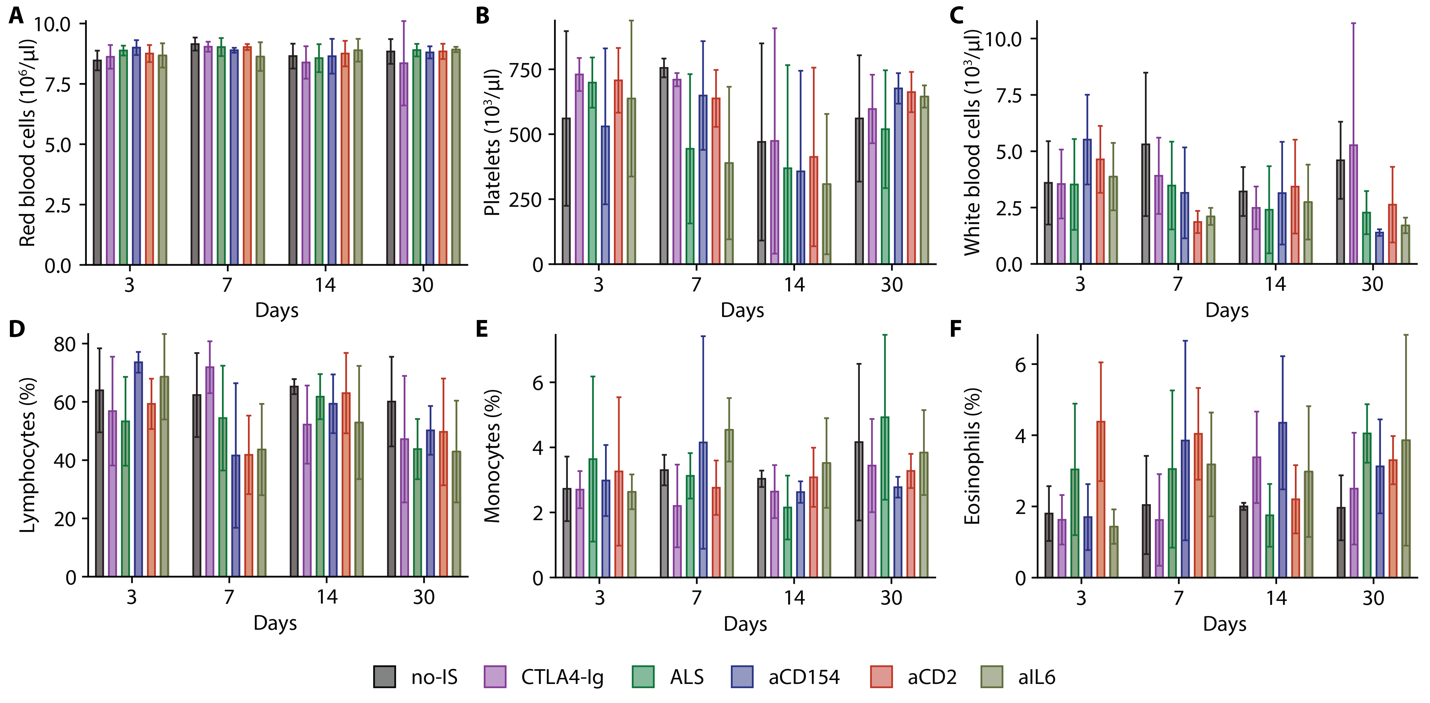


**Figure S5.** Complete blood count. No significant differences were observed across IS groups compared to control group without IS over the 30-day period (n=5/group/timepoint).


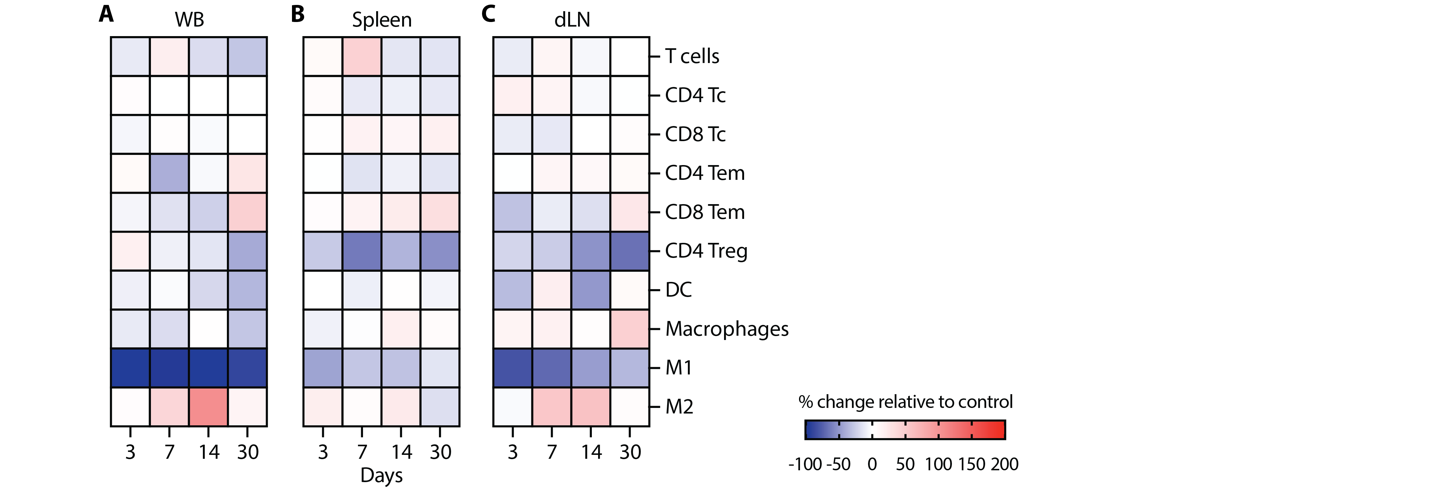


**Figure S6.** Relative change in systemic immune cell populations with CTLA4-Ig local delivery. Flow cytometry data presented as mean percentage change relative to untreated control.


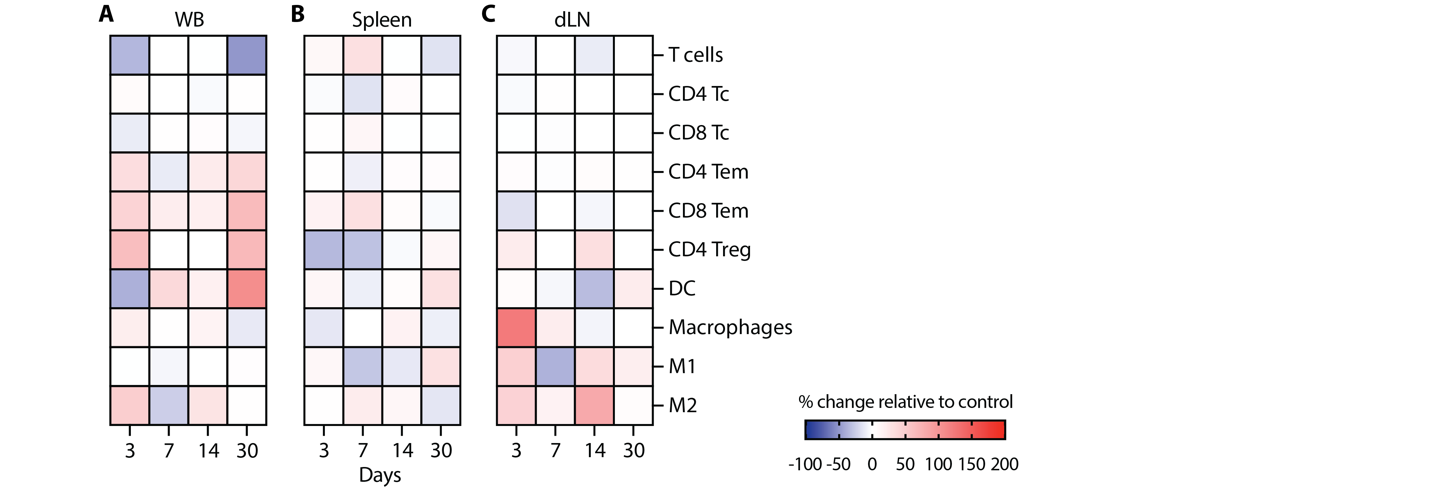


**Figure S7.** Relative change in systemic immune cell populations with ALS local delivery. Flow cytometry data presented as mean percentage change relative to untreated control.

**
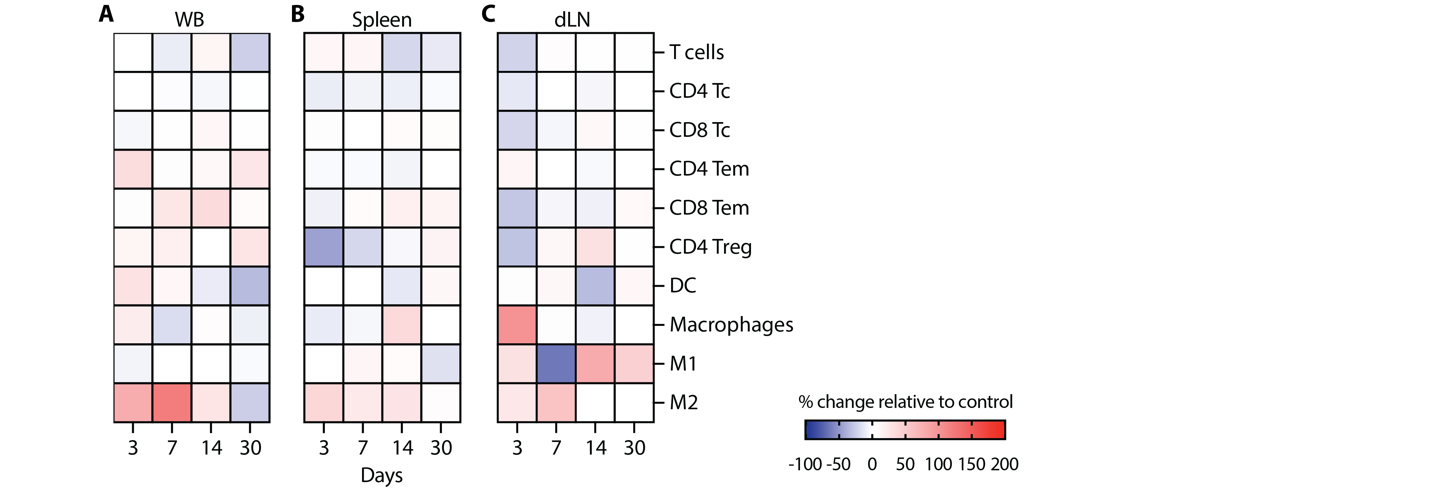
**

**Figure S8.** Relative change in systemic immune cell populations with anti-CD40L local delivery. Flow cytometry data presented as mean percentage change relative to untreated control.


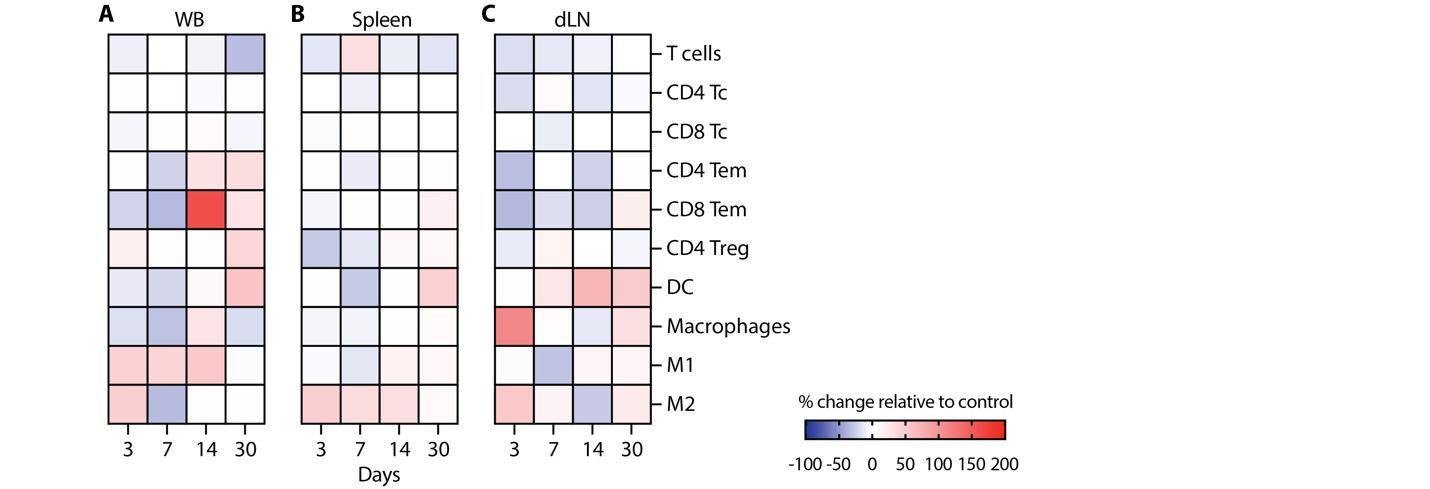


**Figure S9.** Relative change in systemic immune cell populations with anti-CD2 local delivery. Flow cytometry data presented as mean percentage change relative to untreated control.


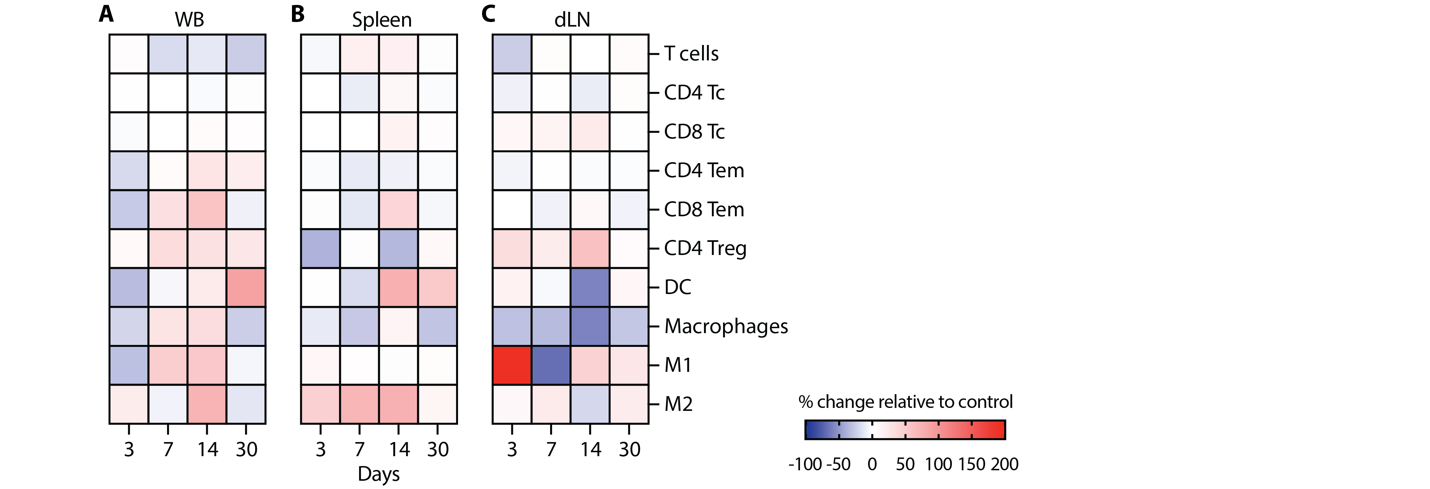


**Figure S10.** Relative change in systemic immune cell populations with anti-IL6 local delivery. Flow cytometry data presented as mean percentage change relative to untreated control.

**
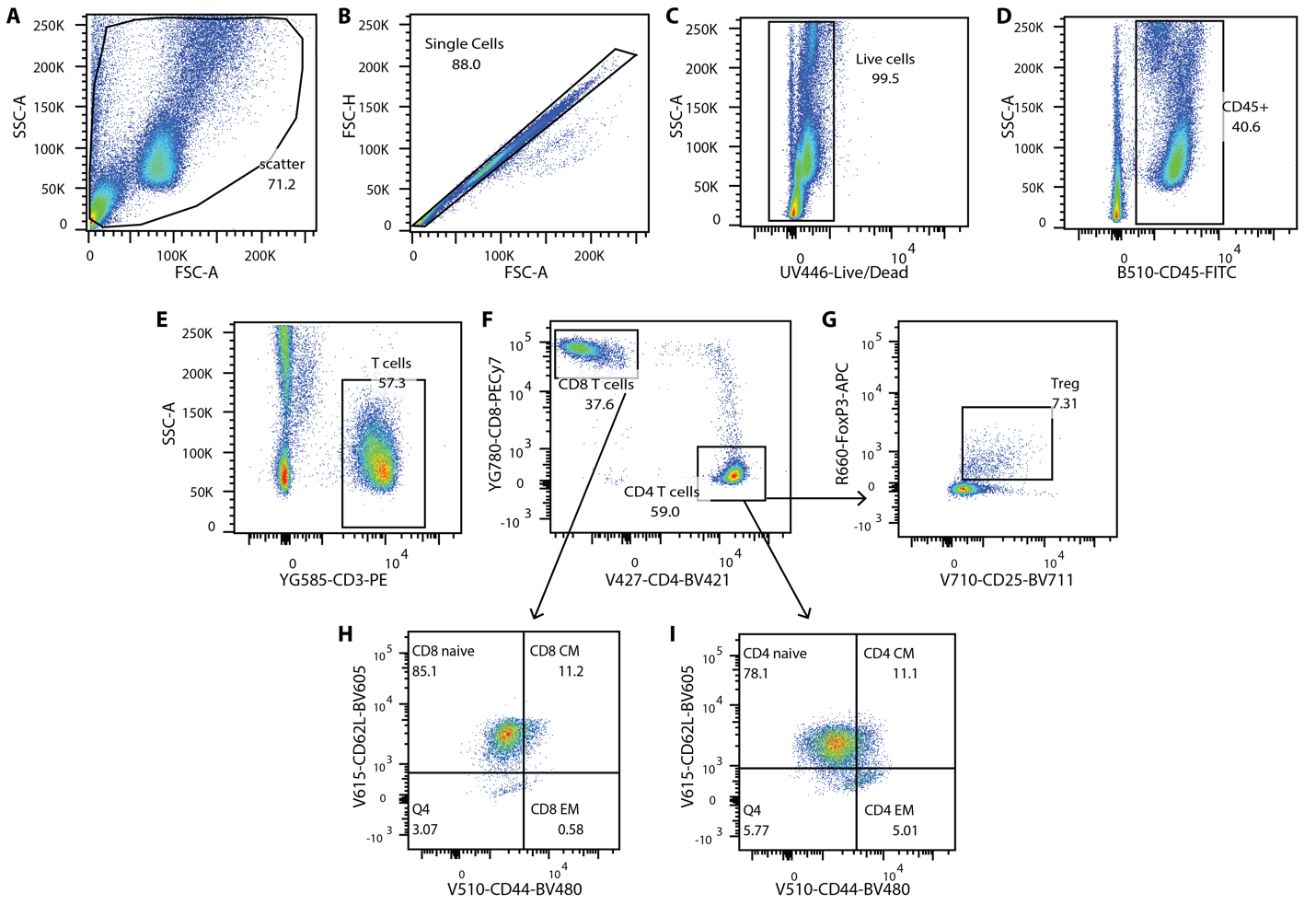
**

**Figure S11.** Gating strategy for flow cytometry lymphoid panel in whole blood. Representative pseudocolor plots are displayed for gating of (A) scatter, (B) singlets, (C) live cells, (D) CD45^+^ cells, (E) T cells, (F) CD8^+^ and CD4^+^ T cells, (G) CD4^+^ Treg, (H) CD8^+^ and (I) CD4^+^ memory T cells. Central memory (CM); effector memory (EM).

**
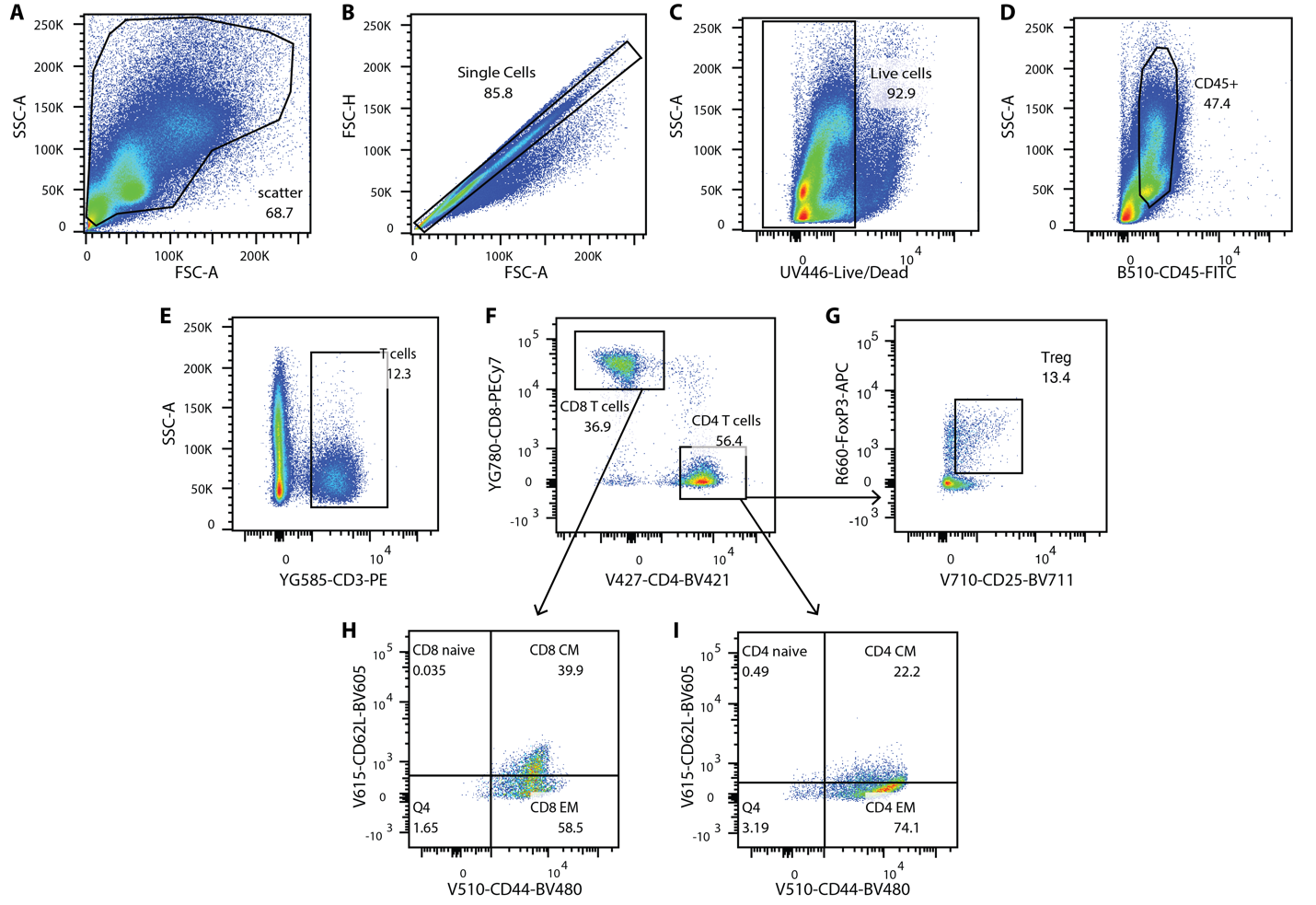
**

**Figure S11 (continued).** Gating strategy for flow cytometry lymphoid panel in spleen. Representative pseudocolor plots are displayed for gating of (A) scatter, (B) singlets, (C) live cells, (D) CD45^+^ cells, (E) T cells, (F) CD8^+^ and CD4^+^ T cells, (G) CD4^+^ Treg, (H) CD8^+^ and (I) CD4^+^ memory T cells. Central memory (CM); effector memory (EM).

**
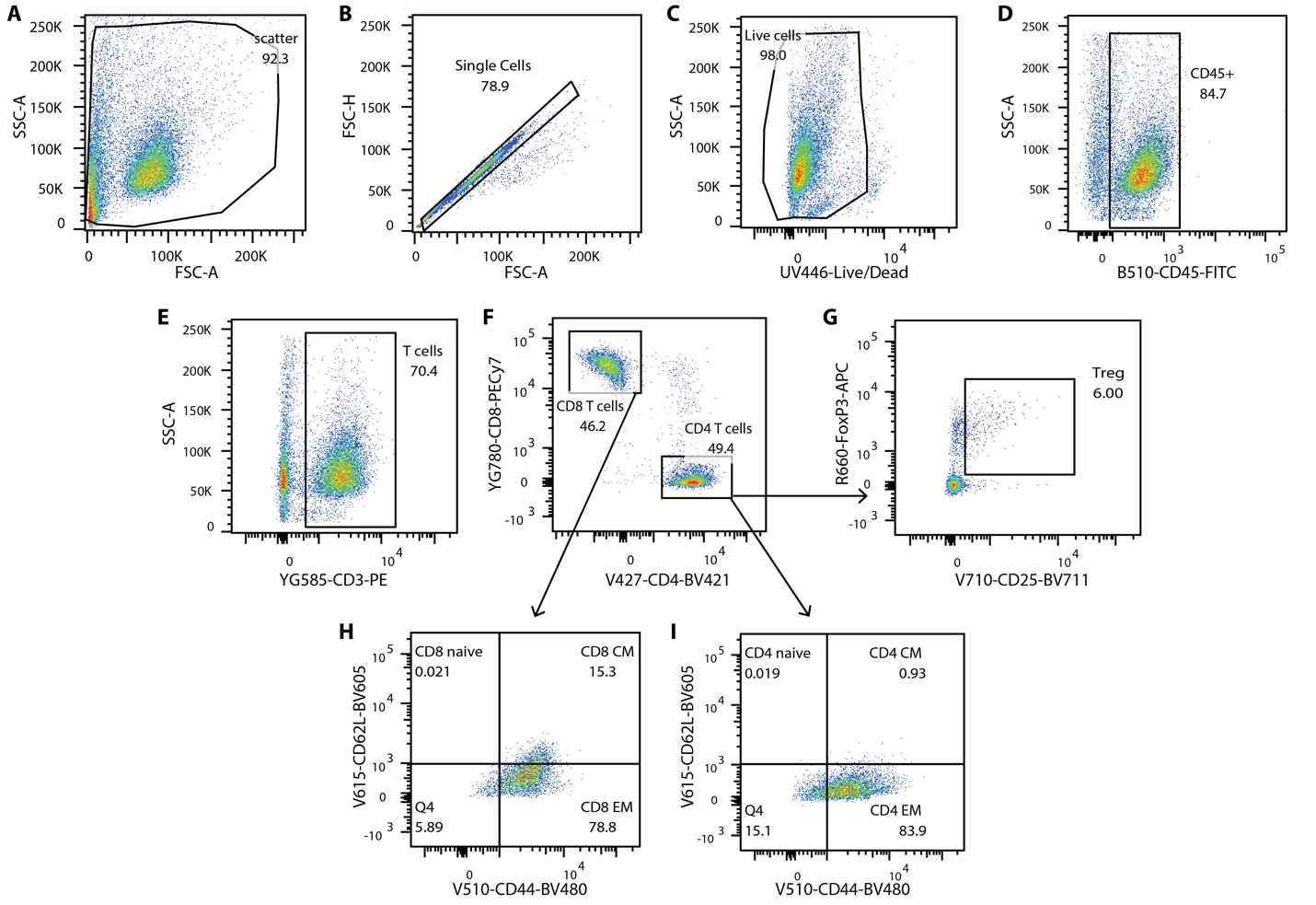
**

**Figure S11 (continued).** Gating strategy for flow cytometry lymphoid panel in draining lymph node. Representative pseudocolor plots are displayed for gating of (A) scatter, (B) singlets, (C) live cells, (D) CD45^+^ cells, (E) T cells, (F) CD8^+^ and CD4^+^ T cells, (G) CD4^+^ Treg, (H) CD8^+^ and (I) CD4^+^ memory T cells. Central memory (CM); effector memory (EM).


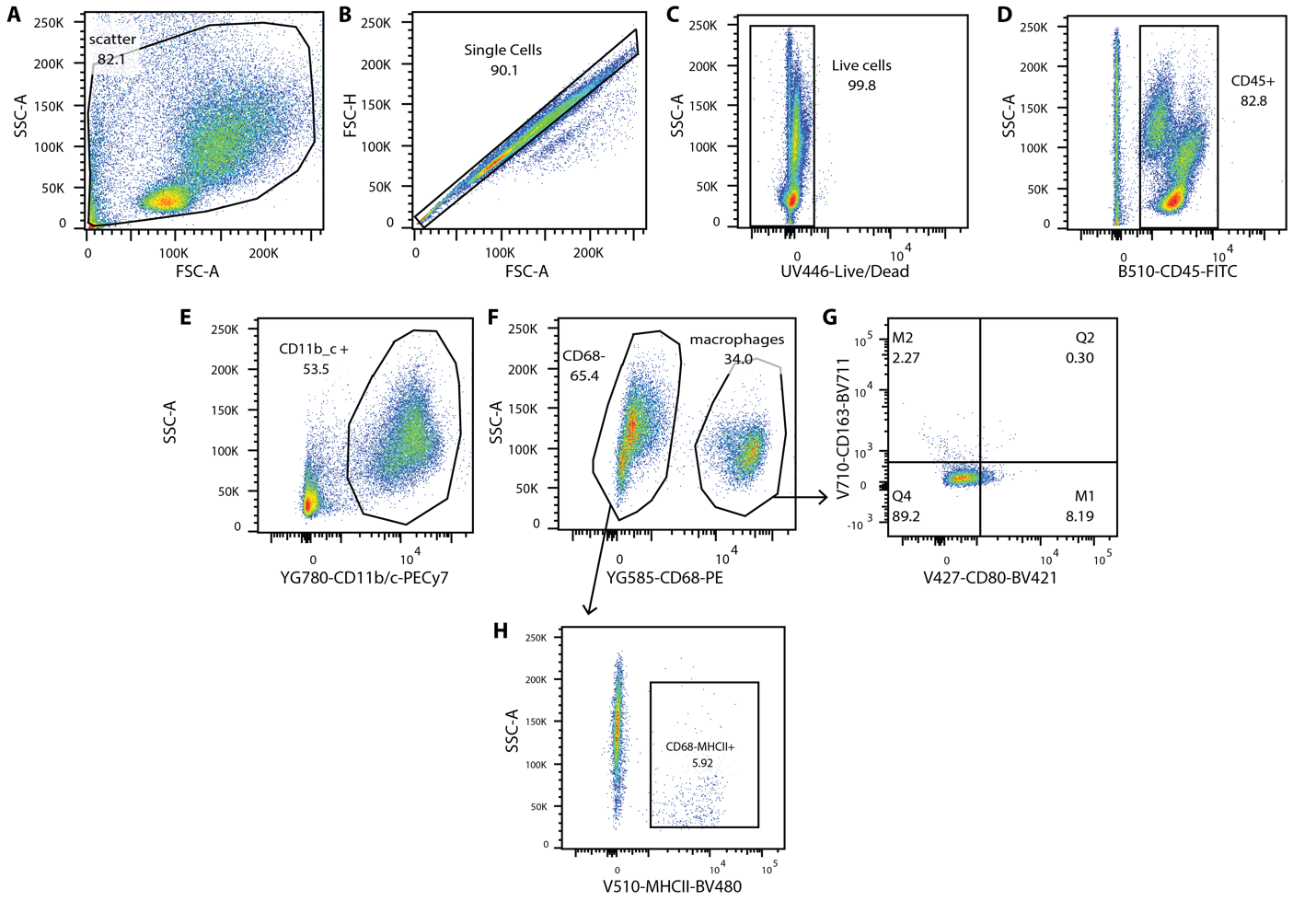


**Figure S12.** Gating strategy for flow cytometry myeloid panel in whole blood. Representative pseudocolor plots are displayed for gating of (A) scatter, (B) singlets, (C) live cells, (D) CD45^+^ cells, (E) CD11b^+^ cells, (F) CD68^+^ (macrophages) and CD68^-^ cells, (G) M1 and M2, (H) CD68^-^MHCII^+^ (dendritic cells).


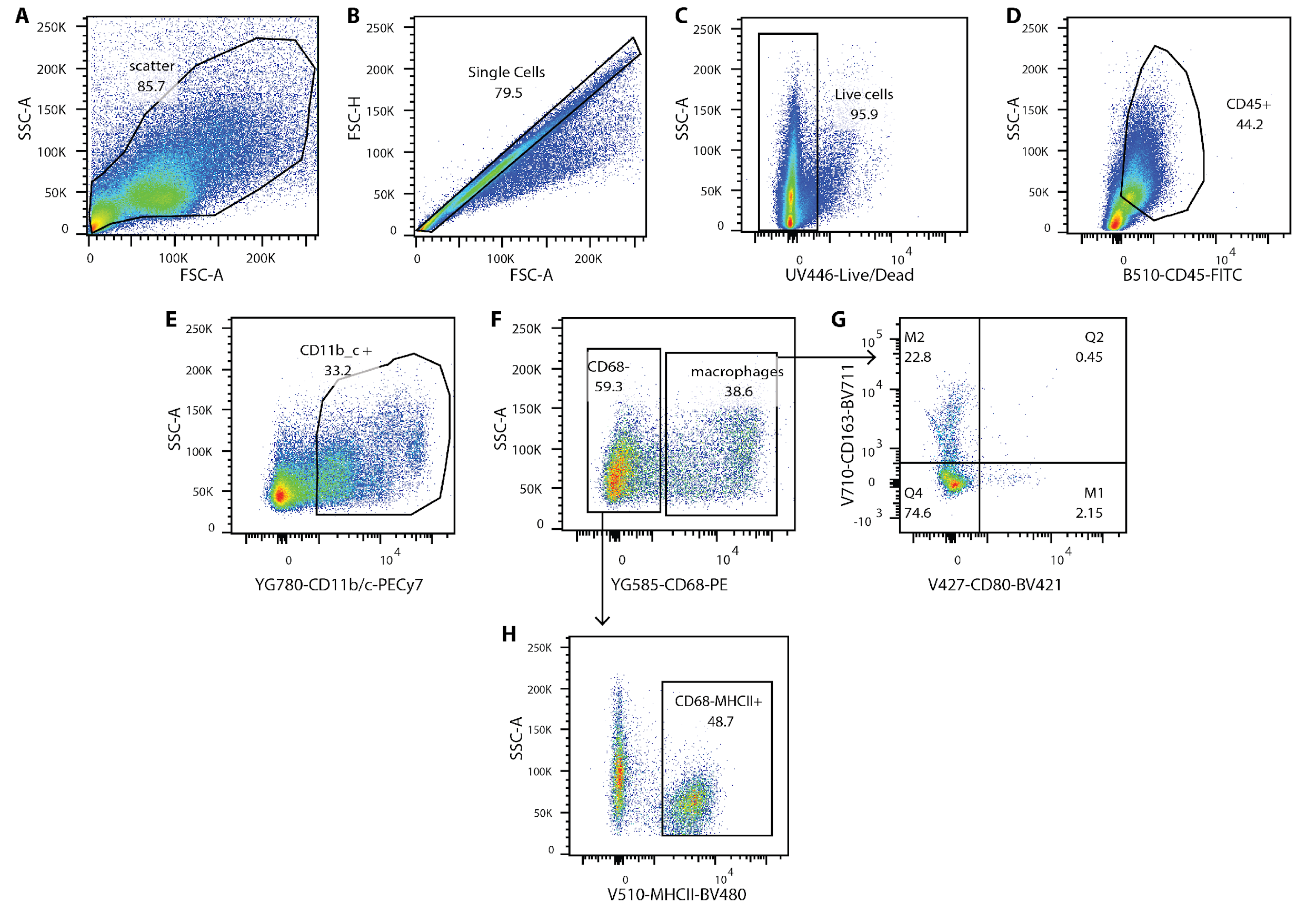


**Figure S12 (continued).** Gating strategy for flow cytometry myeloid panel in spleen. Representative pseudocolor plots are displayed for gating of (A) scatter, (B) singlets, (C) live cells, (D) CD45^+^ cells, (E) CD11b^+^ cells, (F) CD68^+^ (macrophages) and CD68^-^ cells, (G) M1 and M2, (H) CD68^-^MHCII^+^ (dendritic cells).


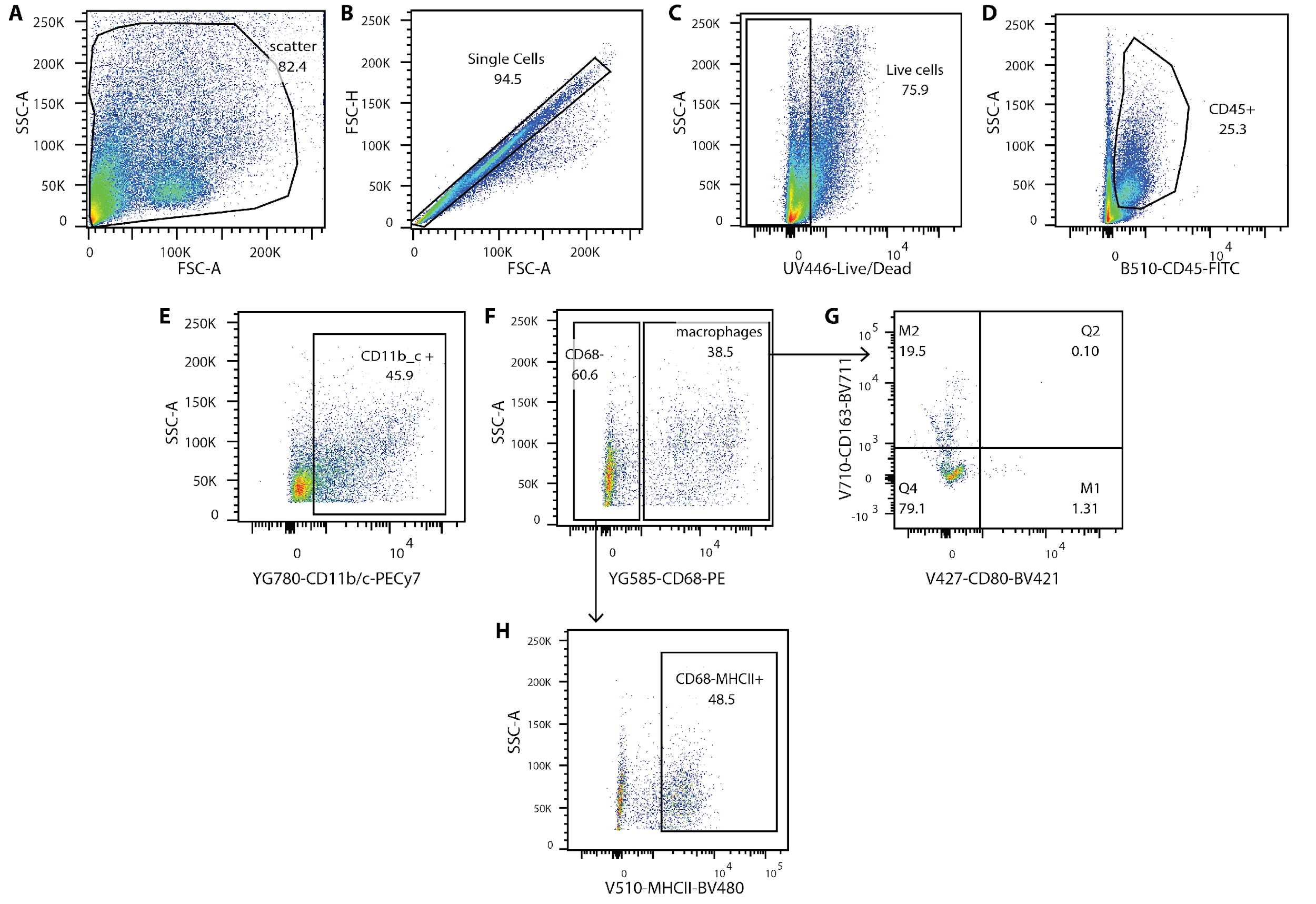


**Figure S12 (continued).** Gating strategy for flow cytometry myeloid panel in draining lymph node. Representative pseudocolor plots are displayed for gating of (A) scatter, (B) singlets, (C) live cells, (D) CD45^+^ cells, (E) CD11b^+^ cells, (F) CD68^+^ (macrophages) and CD68^-^ cells, (G) M1 and M2, (H) CD68^-^MHCII^+^ (dendritic cells).

| Clinical correlate | Dosing | Cmax  [µg ml^-1^] | Cmax,plasma – NICHE  [µg ml^-1^] | Cmax,local – NICHE  [µg ml^-1^] | Reference |
| --- | --- | --- | --- | --- | --- |
| **CTLA4Ig (belatacept)** | 10 mg kg^-1^ IV on Day 1, Day 5, end of Weeks 2, 4, 8, 12; then 5 mg kg^-1^ IV every 4 weeks (maintenance**)** | 10 mg kg^-1^ IV, Week 12 in transplant pts: 247 ± 68  5 mg kg^-1^ IV, Month ≥12 in transplant pts: 139 ± 28 | 22.6 ± 8.0 | 187.0 ± 71.0 | (Food and Drug Administration, 2018; Vincenti et al., 2016) |
| **ATG (Thymoglobulin)** | 1.5 mg kg^-1^ day^-1^ IV for 3-5 days | After first dose: 21.5 (range 10–40)    After last dose: mean 87 (range 23–170) | 4.0 ± 2.1 | 9.0 ± 8.0 | (Brennan et al., 2006; Food and Drug Administration, 2024) |
| **anti-IL6**  **(siltuximab)** | 11 mg kg^-1^ IV every 3 weeks | 332 (42% CV) | 1.4 ± 0.7 | 6.2 ± 1.9 | (Food and Drug Administration, 2019; Rhee et al., 2014) |
| **anti-CD40L (dapirolizumab pegol)** | Phase I study with single doses at 5-60 mg kg^-1^) | 5 mg kg^-1^: 0.7–105  15 mg kg^-1^: 09–121  30 mg kg^-1^: 60–294  60 mg kg^-1^: 47–1157 | 2.1 ± 1.1 | 35.4 ± 7.5 | (Tocoian et al., 2015) |
| **anti-CD2 (siplizumab)** | 0.6 mg kg^-1^ x3 | 16.6 (tested in NHP) | 0.6 ± 0.5 | 7.0 ± 2.0 | (Kawai et al., 2008; Sellberg et al., 2020) |

**Table S1.** Cmax after systemic administration of IS in clinical studies compared to local administration via NICHE.

| Marker | Fluorophore | Clone | Make | Cat No. | Dilution |
| --- | --- | --- | --- | --- | --- |
| Cell Trace Violet | Cell Trace Violet | - | Invitrogen | C34557 | 1:2000 |
| Rat serum | None | - | Invitrogen | 24-5555-94 | None |
| CD45 | FITC | OX-1 | BD Biosciences | 561867 | 1:200 |
| CD3 | BV605 | 1F4 | BD Biosciences | 563949 | 1:200 |
| CD4 | BV510 | OX-35 | BD Biosciences | 740138 | 1:200 |
| CD8a | PerCP | OX8 | BD Biosciences | 558824 | 1:200 |
| CD45RA | PE | OX-33 | BD Biosciences | 551402 | 1:200 |
| CD161 | RB780 | 10/78 | BD Biosciences | 756024 | 1:200 |
| CD25 | BV421 | OX-39 | BD Biosciences | 565608 | 1:200 |
| FOXP3 | APC | FJK-16s | eBioscience | 17-5773-82 | 1:50 |
| IgG2 | APC | eBR2a | Invitrogen | 17-4321-81 | 1:50 |
| LIVE/DEAD | APC-Cy7 | - | Invitrogen | L10119 | 1:1000 |

**Table S2.** Antibodies used for in vitro mixed lymphocyte reaction (MLR).

| Metal | Target | Make | Cat No. | Clone |
| --- | --- | --- | --- | --- |
| Pr141 | CD45 | BD Biosciences | 554875 | OX-1 |
| Sm154 | CD3 | eBioscience | 14-0030-82 | G4.18 |
| Yb176 | CD4 | eBioscience | 14-0040-82 | OX-35 |
| Dy162 | CD8 | eBioscience | 14-0084-82 | OX-8 |
| Dy164 | CD25 | BD Biosciences | 559980 | OX-39 |
| Gd160 | Foxp3 | Biolegend | 353202 | G043H7 |
| Nd148 | CTLA4 | Invitrogen | MA1-70039 | WKH 203 |
| Sm152 | CD44 | BD Biosciences | 554869 | OX-49 |
| Tb159 | CD62L | Invitrogen | MA1-70037 | OX-85 |
| Dy161 | GranZyme B | Biolegend | 372202 | QA16A02 |
| Ho165 | CD161 | Biolegend | 251102 | QA19A15 |
| Gd155 | CD45RA | BD Biosciences | 554882 | OX-33 |
| Gd158 | CD43 | Abcam | ab22351 | W3/13 |
| Nd150 | CD11b/c | Biolegend | 201801 | OX-42 |
| Nd143 | CD11b | Invitrogen | PA5-79532 | Polyclonal |
| Nd142 | MHCII | BD Biosciences | 554926 | OX-6 |
| Sm147 | CD68 | Biolegend | 201002 | QA20A71 |
| Er167 | CD163 | Bio-rad | MCA342GA | ED2 |
| Er168 | CD80 | Biolegend | 200202 | 3H5 |
| Gd156 | CD28 | BD Biosciences | 559982 | JJ319 |

**Table S3.** Markers used for CyTOF analysis.

| Cell population | Gating strategy (population/parent) |
| --- | --- |
| B cells | CD45RA^+^CD3^-^/CD45^+^ |
| Neutrophils | CD3^-^CD11b^+^CD43^hi^CD68^-^/CD45^+^ |
| DC | CD11c^+^CD11b^-^CD68^-^/CD45^+^ |
| Macrophages | CD11b^+^CD11c^-^CD68^+^/CD45 |
| NK cells | CD3^-^CD161^+^/CD45^+^ |
| T cells | CD3^+^/CD45^+^ |
| CD4^+^ T cells | CD3^+^CD4^+^CD8^-^/CD45^+^ |
| CD8^+^ T cells | CD3^+^CD4^-^CD8^+^/CD45^+^ |
| CD4^+^ Tem | CD3^+^CD4^+^CD8^-^CD44^hi^CD62L^lo^/ CD45^+^ |
| CD8^+^ Tem | CD3^+^CD4^-^CD8^+^CD44^hi^CD62L^lo^/CD45^+^ |
| CD4^+^ Tcm | CD3^+^CD4^+^CD8^-^CD44^hi^CD62L^hi^/CD45^+^ |
| CD8^+^ Tcm | CD3^+^CD4^-^CD8^+^CD44^hi^CD62L^hi^/ CD45^+^ |
| CD4^+^ Treg | CD4^+^CD8^-^CD25^+^Foxp3^+^/CD45^+^CD3^+^ |
| CTLA4^+^CD4^+^ T cells | CD4^+^CD8^-^CTLA4^+^/CD45^+^CD3^+^ |
| CD28^+^CD4^+^ T cells | CD4^+^CD8^-^CD28^+^/CD45^+^CD3^+^ |
| CD28^+^CD8^+^ T cells | CD4^-^CD8^+^CD28^+^/CD45^+^CD3^+^ |
| GranzymeB^+^CD8^+^ T cells | CD4^-^CD8^+^GranzB^hi^/CD45^+^CD3^+^ |
| M1 macrophages | CD80^+^/CD45^+^CD11b^+^CD11c^-^CD68^+^ |
| M2 macrophages | CD163^+^/CD45^+^CD11b^+^CD11c^-^CD68^+^ |

**Table S4.** CyTOF gating strategy

| Marker | Fluorophore | Clone | Make | Cat No. | Dilution |
| --- | --- | --- | --- | --- | --- |
| FC blocker (aCD32) | None | D34-485 | BD Biosciences | 550270 | 1:500 |
| CD45 | FITC | OX-1 | eBioscience | 11-0461-82 | 1:250 |
| CD3 | PE | G4.18 | eBioscience | 12-0030-82 | 1:300 |
| CD4 | BV421 | OX-35 | BD Biosciences | 740040 | 1:300 |
| CD8a | PE-Cy7 | OX8 | eBioscience | 25-0084-82 | 1:300 |
| CD44 | BV480 | OX-49 | BD Biosciences | 746710 | 1:300 |
| CD62L | BV605 | HLR1 | BD Biosciences | 743149 | 1:300 |
| CD25 | BV711 | OX-39 | BD Biosciences | 742756 | 1:300 |
| FOXP3 | APC | FJK-16s | eBioscience | 17-5773-82 | 1:50 |
| CD11b/c | PE-Cy7 | OX-42 | Biolegend | 201818 | 1:100 |
| RT1B (MHCII) | BV480 | OX-6 | BD Biosciences | 746350 | 1:150 |
| CD80 | BV421 | 3H5 | BD Biosciences | 743863 | 1:200 |
| CD163 | BV711 | HIS36 | BD Biosciences | 744185 | 1:200 |
| CD68 | PE | QA20A71 | Biolegend | 201004 | 1:50 |
| Viability Dye | UV Blue | - | Invitrogen | L23105 | 1:125 |

**Table S5.** Antibodies used for in vivo study flow cytometry lymphoid and myeloid panel.

**References**

Brennan, C. D., Daller, A. J., Lake, D. K., Cibrik, D., & Castillo, D. D. (2006). Rabbit Antithymocyte Globulin versus Basiliximab in Renal Transplantation. *New England Journal of Medicine*, *355*(19), 1967-1977. https://doi.org/https://doi.org/10.1056/nejmoa060068

Food and Drug Administration. (2018). *Label for NULOJIX (belatacept) (FDA)*. Retrieved from https://www.accessdata.fda.gov/drugsatfda_docs/label/2018/125288s075lbl.pdf

Food and Drug Administration. (2019). *Label for SYLVANT (siltuximab) (FDA)*. Retrieved from https://www.accessdata.fda.gov/drugsatfda_docs/label/2019/125496s018lbl.pdf

Food and Drug Administration. (2024). *THYMOGLOBULIN (anti-thymocyte globulin [rabbit]) (FDA)*. Retrieved from https://www.fda.gov/media/74641/download

Kawai, T., Cosimi, B. A., Spitzer, R. T., Tolkoff-Rubin, N., Suthanthiran, M., Saidman, L. S.,…Sachs, H. D. (2008). HLA-Mismatched Renal Transplantation without Maintenance Immunosuppression. *New England Journal of Medicine*, *358*(4), 353-361. https://doi.org/https://doi.org/10.1056/nejmoa071074

Rhee, V. F., Wong, S. R., Munshi, N., Rossi, J.-F., Ke, X.-Y., Fosså, A.,…Casper, C. (2014). Siltuximab for multicentric Castleman's disease: a randomised, double-blind, placebo-controlled trial. *The Lancet Oncology*, *15*(9), 966-974. https://doi.org/https://doi.org/10.1016/s1470-2045(14)70319-5

Sellberg, F., Berglund, D., Binder, C., Hope, J., Fontenot, J., Griesemer, A.,…Berglund, E. (2020). Pharmacokinetic and pharmacodynamic study of a clinically effective anti‐CD2 monoclonal antibody. *Scandinavian Journal of Immunology*, *91*(1). https://doi.org/10.1111/sji.12839

Tocoian, A., Buchan, P., Kirby, H., Soranson, J., Zamacona, M., Walley, R.,…Oliver, R. (2015). First-in-human trial of the safety, pharmacokinetics and immunogenicity of a PEGylated anti-CD40L antibody fragment (CDP7657) in healthy individuals and patients with systemic lupus erythematosus. *Lupus*, *24*(10), 1045-1056. https://doi.org/https://doi.org/10.1177/0961203315574558

Vincenti, F., Rostaing, L., Grinyo, J., Rice, K., Steinberg, S., Gaite, L.,…Larsen, P. C. (2016). Belatacept and Long-Term Outcomes in Kidney Transplantation. *New England Journal of Medicine*, *374*(4), 333-343. https://doi.org/https://doi.org/10.1056/nejmoa1506027
